# In Silico Characterization of the RCC1 Family and the UVR8 Gene in *Chenopodium quinoa* Willd.

**DOI:** 10.3390/ijms262311657

**Published:** 2025-12-01

**Authors:** Jean Carlo Paredes Malca, Michell Maheba Fuentes Apaza, María Rosario Elsa Valderrama-Valencia, Roxana Bardales Álvarez, Eloy Condori Mamani, Sandro Jhonatan Condori-Pacsi

**Affiliations:** Laboratorio de Recursos Genéticos y Genética Molecular, Universidad Nacional de San Agustín, Arequipa 04001, Peru; jparedesm@unsa.edu.pe (J.C.P.M.); mfuentesa@unsa.edu.pe (M.M.F.A.); mvalderramav@unsa.edu.pe (M.R.E.V.-V.); rbardales@unsa.edu.pe (R.B.Á.); eloy.cm@usp.br (E.C.M.)

**Keywords:** *Chenopodium quinoa*, UVR8, RCC1, bioinformatic, photoreceptor UV-B, comparative modeling, abiotic stress

## Abstract

Quinoa (*Chenopodium quinoa* Willd.), an Andean crop with exceptional nutritional value, thrives in ecosystems exposed to intense ultraviolet-B (UV-B) radiation; yet the molecular mechanisms underlying its photoreception remain largely unknown. The UV Resistance locus 8 (UVR8) protein, a member of the Regulator of Chromosome Condensation 1 (RCC1) family, is the primary UV-B photoreceptor in plants. Here, we report the first in silico characterization of the *RCC1* gene family in *C. quinoa*, aimed at identifying and structurally analyzing UVR8 homologs. Genomic analysis uncovered 40 *CqRCC1* genes, exhibiting extensive structural diversity. Phylogenetic reconstruction identified two proteins, CqRCC1_20 and CqRCC1_23, as the closest homologs of AtUVR8 from *Arabidopsis thaliana*. Homology modeling revealed that CqRCC1_20 maintains the canonical seven-bladed β-propeller architecture of UVR8, whereas CqRCC1_23 carries a deletion leading to a six-bladed structure. Both isoforms retain the critical tryptophan residues (W233, W285, W337) and the C-terminal Valine-Proline (VP) motif required for photoperception and Constitutive Photomorphogenic 1 (COP1) interaction. Notably, the CqRCC1_23 model predicts fewer hydrogen bonds at the dimer interface and structural alterations at key regulatory interaction sites. Collectively, these results indicate that quinoa harbors functionally conserved UVR8 isoforms with structural divergence, such as CqRCC1_23, which may influence photoreceptor stability and enable a sustained UV-B response, potentially conferring an adaptive advantage in high-radiation environments.

## 1. Introduction

Climate change has emerged as one of the most pressing threats to global food security, not only by altering water and temperature availability but also by intensifying the exposure of crops to elevated ultraviolet-B (UV-B; 280–315 nm) radiation. Although UV-B represents only a minor fraction of the solar spectrum that reaches the Earth’s surface, its physiological effects are disproportionately severe, including inhibition of photosynthesis, chlorophyll depletion, DNA damage, and reductions in biomass and yield [1,2]. Such stressors compromise both crop productivity and the nutritional quality of sensitive agricultural species.

Among stress-resilient crops, *Chenopodium quinoa* Willd. stands out as a strategic Andean species with exceptional nutritional properties. Often described as one of the most complete grains, quinoa provides a balanced composition of essential amino acids—particularly lysine (5.1–6.4%) and methionine (0.4–1.0%)—along with a wide range of vitamins and minerals, while being naturally gluten-free [3]. Its remarkable phenotypic plasticity enables growth from sea level to elevations exceeding 4500 m a.s.l., under conditions of drought, salinity, cold, heat, and high radiation [4,5].

Plant responses to UV-B are primarily mediated by the photoreceptor UV Resistance locus 8 (UVR8), a member of the RCC1 (Regulator of Chromosome Condensation 1) family characterized by β-propeller repeats. Distinct from other photoreceptors, UVR8 does not rely on external chromophores but instead absorbs UV-B photons through conserved tryptophan residues [6,7]. In *Arabidopsis thaliana*, with 24 *RCC1* family genes, UVR8 transitions from an inactive homodimer to an active monomer upon UV-B exposure, subsequently interacting with COP1 (Constitutive Photomorphogenic 1) and inducing the expression of photoprotective regulators such as HY5 (Elongated Hypocotyl 5) and WRKY36 (WRKY DNA-Binding Protein 36) [8,9].

Despite the growing importance of quinoa in climate change scenarios and its potential as a model species, the UVR8 homolog in *C. quinoa* remains uncharacterized. This knowledge gap constrains our understanding of the molecular basis of its tolerance to high radiation and its potential application in crop improvement. Here, we present the first structural characterization of CqUVR8 using bioinformatics and homology modeling, evaluating its conservation relative to AtUVR8 and the presence of key functional residues. This work provides a foundation for future efforts to develop quinoa varieties with improved UV-B tolerance, thereby supporting resilient and sustainable agriculture under changing climatic conditions.

## 2. Results

### 2.1. Identification of the RCC1 Family in Chenopodium quinoa

From the analysis of the *C. quinoa* genome, 40 coding sequences containing the conserved RCC1 domain were identified. For nomenclature, the prefix “Cq” was assigned, followed by “RCC1” and a progressive number from 1 to 40, reflecting their order of detection. As shown in Table 1, the identified proteins exhibited wide variability in length and molecular weight, with CqRCC1_34 being the largest protein at 1295 amino acids (140.62 kDa), whereas CqRCC1_36 was the shortest, comprising 147 amino acids (16.11 kDa). This structural diversity suggests a potential functional specialization among RCC1 family members in quinoa.

The isoelectric point (pI) values ranged widely, from 4.73 (CqRCC1_15) to 9.32 (CqRCC1_40), with 26 proteins classified as acidic and 14 as basic (Table 1). Such variability in pI may reflect differences in stability, protein interactions, or subcellular localization. Regarding their predicted distribution, the 40 CqRCC1 proteins exhibited diverse and often multiple subcellular localizations (Table 1). Most proteins were predicted to localize in the endoplasmic membrane (25), nucleus (20), and cytoplasm (12). Notably, some isoforms showed more specialized localizations: CqRCC1_3 was the only one predicted in both chloroplast and mitochondria, while a group of five proteins, including CqRCC1_11, CqRCC1_12, and CqRCC1_16, were associated with the peroxisome, suggesting possible involvement in redox metabolism or signaling functions.

### 2.2. Phylogenetic Analysis of CqRCC1

**Figure 1:**
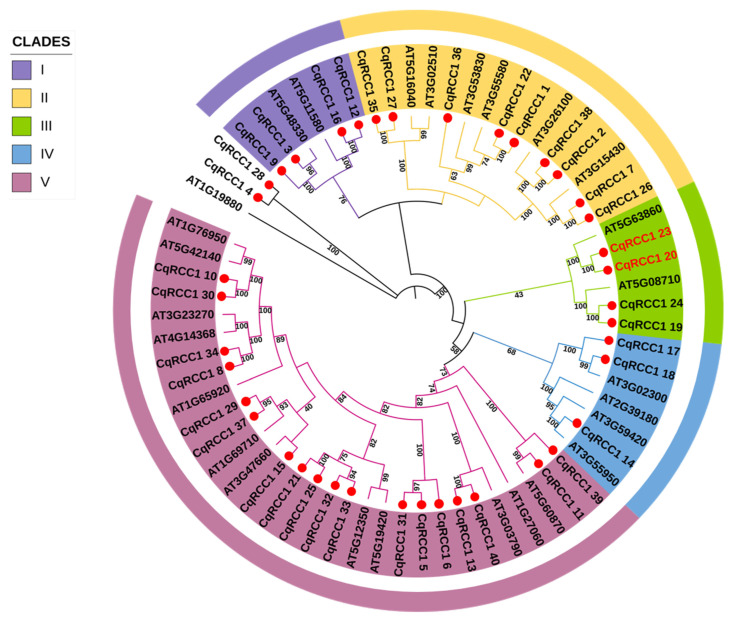
Phylogenetic tree of the RCC1 genes of *Chenopodium* quinoa and *Arabidopsis* thaliana, constructed using IQ-TREE v2.3.5 software, using the maximum likelihood method and the JTT + F + I + G4 model, with a bootstrap value of 1000. The genes are grouped into five subfamilies: Clade I (purple), Clade II (yellow), Clade III (green), Clade IV (blue) and Clade V (pink). The red circles represent the RCC1 genes of *C. quinoa*, and the names in red font represent the isoforms related to AtUVR8.

The 40 RCC1 proteins from *C. quinoa* and the 27 proteins from *A. thaliana* were aligned to generate a phylogenetic tree using the maximum likelihood method with the Jones-Taylor-Thornton (JTT + F + I) substitution method plus gamma (+4G) distribution. Figure 1 shows that the CqRCC1 family formed five clades (I–V), with 3, 9, 4, 3, and 18 members, respectively, clade V being the largest. Furthermore, CqRCC1_20 and CqRCC1_23 clustered with At5G63860, which corresponds to the AtUVR8 gene, a key component in the UV-B radiation response.

### 2.3. Structural and Genomic Analysis of CqRCC1

The 40 coding sequences of RCC1 proteins in *C. quinoa* were analyzed to characterize gene structure and conserved domain composition. Functional annotation via the NCBI CDD revealed the presence of 2 to 7 RCC1 domains per protein, highly conserved across all analyzed sequences, suggesting functional conservation at the structural level. The proteins CqRCC1_20 and CqRCC1_23, closely related to UVR8, exhibited representative domain configurations: CqRCC1_20 contained seven complete RCC1 domains, while CqRCC1_23 also presented seven RCC1 domains. This arrangement may be linked to differences in protein–protein interactions or light signal responses.

Conserved motif analysis using MEME identified ten major motifs within RCC1 proteins. Motifs 3, 6, and 7 were exclusive to proteins in Clade I. Interestingly, some motifs showed similarity to FYVE and BRX-like sequences, which are involved in lipid and metal ion binding, respectively. Additionally, CqRCC1_15 was the only protein containing a 14-3-3-like motif, typically implicated in protein–protein interactions. However, these elements were not annotated as structural domains by NCBI CDD or other structural databases; thus, they should be considered putative functional motifs pending experimental validation.

**Figure 2:**
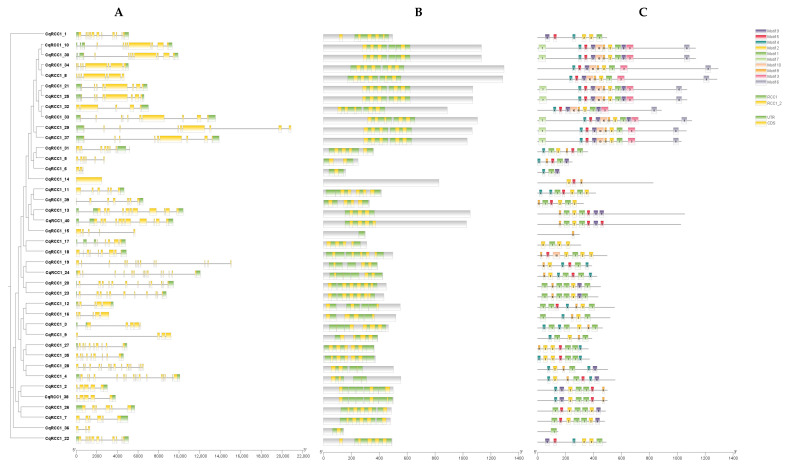
Gene structure and motif analysis of the RCC1 family in *C. quinoa*. (**A**) Conserved sequence motifs of CqRCC1 identified by MEME, with scale in base pairs (bp). (**B**) Conserved domain analysis of CqRCC1 using the NCBI CDD, with scale in amino acids (aa) number. (**C**) Gene organization of *CqRCC1* transcripts: exons (yellow boxes), introns (black lines), and untranslated regions (green boxes), with scale in amino acids (aa) number.

At the genomic level, the *CqRCC1* gene structures displayed marked variability in exon numbers, ranging from 2 to 17. *CqRCC1_4* contained the highest number of exons (17), whereas *CqRCC1_6* was the shortest (2). Overall, 77.5% of the analyzed genes contained between 4 and 10 exons, indicating a moderate degree of structural conservation within the family. This variability suggests diverse functional evolution that may underlie the specialization of CqRCC1 subfamilies (Figure 2).

### 2.4. Multiple Sequence Alignment of CqRCC1

The multiple sequence alignment of the 40 CqRCC1 proteins (Supplementary Figure S1) revealed strong conservation within the central RCC1 domains, composed of β-propeller repeats (Blades 1–7). These regions coincide with key structural sites for protein–protein interaction, suggesting functional preservation among isoforms. By contrast, the N- and C-terminal regions exhibited greater variability, likely associated with functional diversification and specific adaptation processes.

**Figure 3:**
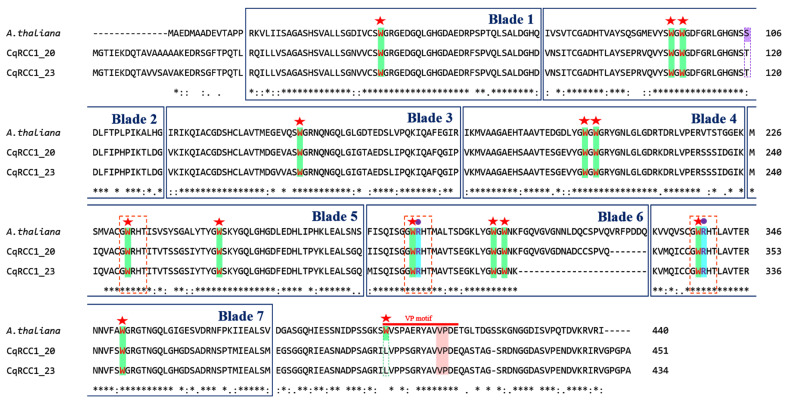
Alignment of amino acid sequences of AtUVR8, CqRCC1_20, and CqRCC1_23. The blue color rectangles define the seven blades of the β-helix. The red stars indicate the 14 conserved tryptophan (W) residues. The red boxes highlight the three conserved ‘GWRHT’ motifs, where the tryptophan triad (W233, W285, and W337) is found, and the purple dots indicate the two arginine residues (R286 and R338). The red line represents the C27 region at the C-terminal end, where the VP motif is located, and the red background highlights the valine and proline residues within this motif. The purple box highlights residues that form hydrogen bonds required for dimer conformation, while the green box indicates the substitution of the 14th tryptophan with leucine in quinoa. The asterisk (*) represents fully conserved residues, the colon (:) indicates amino acids with similar chemical properties, the dot (.) denotes residues without similar chemical properties, and hyphens (-) represent gaps.

Phylogenetic analysis (Figure 1) placed CqRCC1_20 and CqRCC1_23 within Clade II, clustering with their closest homolog in *A. thaliana* (At5G638860). The multiple sequence alignment (Figure 3) confirmed that both isoforms retain all seven RCC1 domains, including the characteristic GWRHT motif in β-blades 5, 6, and 7, a defining feature of UVR8. They also conserve the key photoreceptive residues (W94, W233, W285, and W337) and the basic residues (R286, R338) required for homodimer stabilization. In total, CqRCC1_20 and CqRCC1_23 were found to contain 13 tryptophan (W) residues, which follow a conserved positional pattern comparable to Arabidopsis UVR8, which possesses 14 tryptophan residues.

Finally, both CqRCC1_20 and CqRCC1_23 contained the C-terminal Val–Pro (VP) motif characteristic of UVR8, highly conserved relative to the *A. thaliana* reference sequence. In this model species, the VP motif constitutes the direct interaction site with COP1 and the repressors RUP1 and RUP2, which are key regulators of UVR8 re-dimerization into its inactive form after UV-B exposure. The alignment confirmed that this motif is conserved in sequence and relative position in both quinoa isoforms, indicating preservation of this essential structural feature within the UVR8 family.

### 2.5. Cis-Regulatory Element (CRE) Analysis

**Figure 4:**
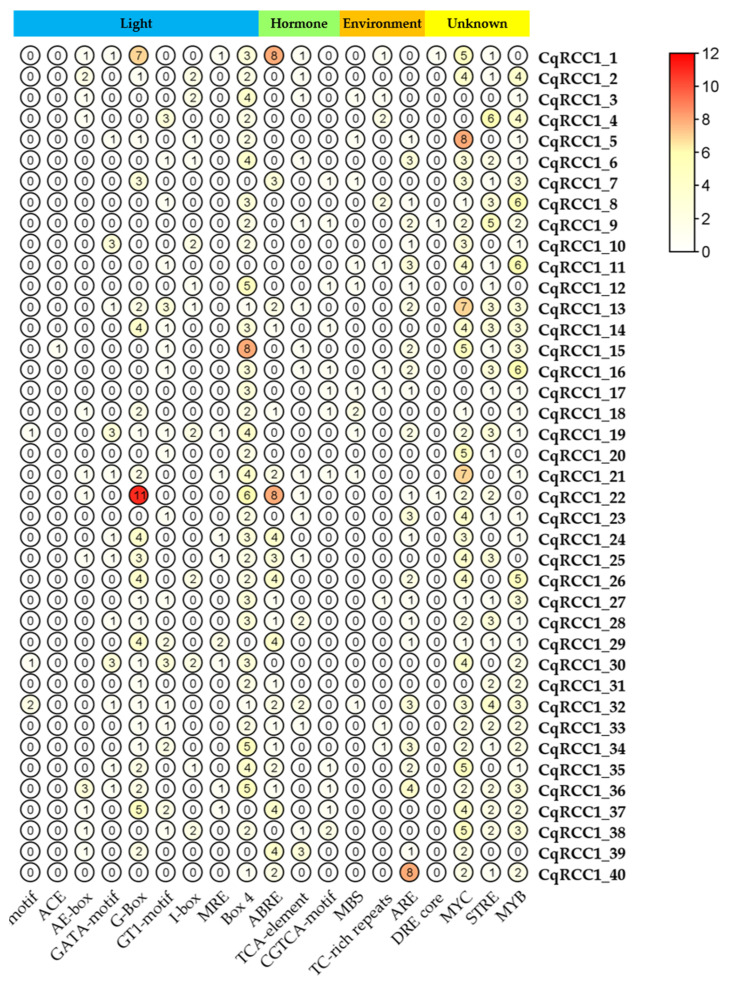
Distribution of cis-regulatory elements (CREs) in the promoter regions of *CqRCC1* genes. CREs detected in each promoter were categorized according to their predicted functions, including hormone-responsive, light-responsive, environment-responsive, and unknown elements. Each circle represents the number of occurrences of a given CRE, with color intensity proportional to the corresponding score. The correspondence between color intensity and score is shown in the scale bar on the right.

Cis-regulatory elements (CREs) were identified within the 1500 bp upstream promoter regions of *C. quinoa RCC1* genes. The detected motifs were grouped into six functional categories: light responsiveness, hormonal regulation, abiotic stress response, development, core promoter elements, and transcription factor binding sites. A summary of 19 common plant cis-motifs is presented in Figure 4, displayed as a color gradient reflecting their relative abundance across sequences.

Notably, the promoters of *CqRCC1_20* and *CqRCC1_23* were enriched in elements associated with UV-B perception and UVR8-mediated signaling pathways, such as GT1-motif, Box 4, and I-box. These motifs are recognized by transcription factors, including HY5, which is activated following UV-B exposure [10,11]. Additionally, motifs linked to abiotic stress responses were also identified, such as ABRE (ABA signaling) and MBS (dehydration response), suggesting a potential integration of light and hormonal signals in the activation of photoprotective pathways.

Remarkably, *CqRCC1_23* harbored three ARE-like motifs, which are annotated in MEME/PlantCARE as putative antioxidant-responsive elements. These are regulatory sequences known to mediate transcriptional activation under oxidative stress conditions in various organisms [12]. Although their functional role in plants is not fully characterized, their presence may indicate potential responsiveness to UV-B–induced photooxidative stress. Collectively, these regulatory patterns indicate that *CqRCC1_20* and *CqRCC1_23* share cis-regulatory features with *UVR8*-controlled genes, reinforcing their putative role in specialized photoprotective mechanisms.

### 2.6. Structural Modeling of CqUVR8

Structural models of CqRCC1_20 and CqRCC1_23 were generated through homology modeling using the crystal structure of the *A. thaliana* UVR8 homodimer (PDB: 4D9S) as a template. This reference structure was selected because AtUVR8 functions physiologically as a homodimer and shows high sequence similarity to the *C. quinoa* proteins, allowing accurate reconstruction of the quaternary arrangement and domain organization. Both sequences were selected based on their clustering in Clade II of the phylogenetic tree (Figure 3) and the conservation of key residues associated with UVR8 functionality.

**Figure 5:**
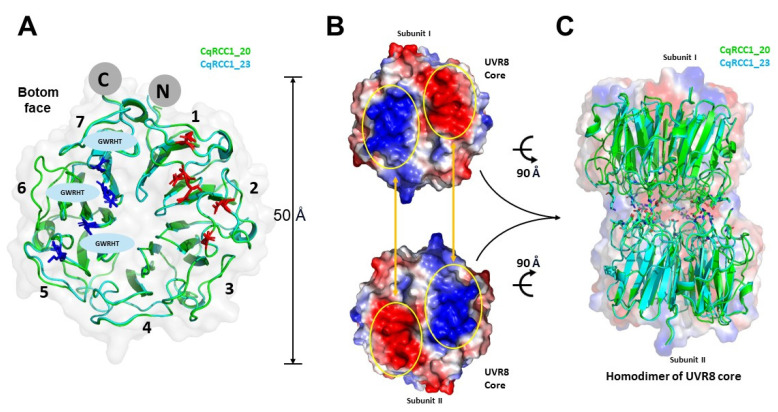
Structural basis of UVR8 homodimer formation in *C. quinoa*. (**A**) The lower surface of the UVR8 homodimer comprises seven β-strand blades, prominently featuring electrostatic patches enriched in positively charged residues (blue) and negatively charged residues (red). These complementary charge distributions are critical for stabilizing intermolecular interactions. The conserved “GWRHT” motif is localized within β-blades 5, 6, and 7. (**B**) Electrostatic surface representation of the lower faces of UVR8 subunits. (**C**) Homodimer formation of quinoa UVR8 mediated by hydrogen bonds stabilized by complementary surface charge patches.

The CqRCC1_23 and CqRCC1_20 models obtained GMQE scores of 0.89 and 0.88, respectively, and QMEAN values of 0.88 ± 0.05 in both cases, indicating high structural quality and reliability. The three-dimensional model of CqRCC1_20 displayed the canonical UVR8 architecture, consisting of seven β-propeller blades arranged radially. In contrast, CqRCC1_23 lacked one β-blade due to a deletion spanning residues F318–Q334 (Figure 3 and Figure 5A), potentially compromising global conformational stability and dimerization capacity. Nevertheless, both proteins retained the functional GWRHT motif between β-blades 5 and 7, which is implicated in UV-B signal perception and transduction, suggesting the preservation of photoreceptor potential.

Electrostatic analysis revealed complementary charge patches on the lower surfaces of both modeled proteins (Figure 5B). An acidic region composed of Glu57, Asp58, and Glu67 (blade 1), together with Asp109 and Asp111 (blade 2), and a basic region comprising Lys266 (blade 5) and Arg299 (blade 6) were identified in both models, in addition to Arg345 and Arg361 (blade 7) in CqRCC1_20, and Arg328 and Arg344 (blade 7) in CqRCC1_23. These residues, conserved in both sequences, are spatially arranged to establish stabilizing interactions through charge complementarity, thereby promoting homodimer formation in a manner analogous to UVR8 from *A. thaliana* [13,14].

**Figure 6:**
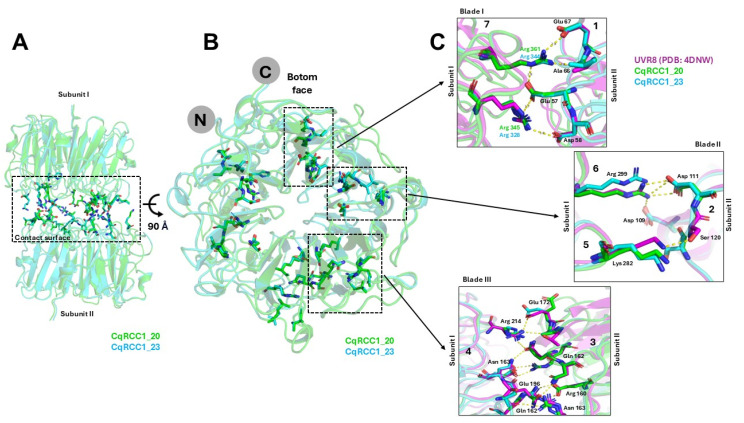
Conserved residues involved in subunit interactions of quinoa UVR8. (**A**) Interaction interface (black box) showing hydrogen bonds between monomers CqRCC1_20 (green) and CqRCC1_23 (cyan). (**B**) Residues from one subunit involved in homodimer interactions are highlighted with black boxes. Arrows point to the regions subsequently magnified in panel (**C**). (**C**) Comparison of hydrogen bonds between key residues, using *A. thaliana* UVR8 (PDB: 4DNW, violet) as a reference structure against CqRCC1_20 and CqRCC1_23 models across blades (numbers indicate the corresponding β-propeller blades) between subunits. Hydrogen bonds are represented as yellow dashed lines.

Structural comparison of the AtUVR8 homodimer (PDB: 4DNW) with the quinoa models indicated a contact surface of approximately 2566 Å^2^ (Figure 6A). In this context, Glu57, Asp58, and Glu67 (blade 1) of one subunit acted as hydrogen bond acceptors from Arg345 and Arg361 in CqRCC1_20, and from Arg328 and Arg344 (blade 7) in CqRCC1_23 of the opposite subunit. Additionally, Asp109 and Asp111 (blade 2) mediated four hydrogen bonds with Arg299 (blade 6) of the complementary subunit (Figure 6B,C). Symmetrical interactions were also identified, in which Gln162, Glu172, and Glu196 acted as acceptors of ten hydrogen bonds donated by Arg214, Asn163, and Arg160 of the opposite subunit (Figure 6C).

The models revealed specific substitutions with potential impact on dimerization dynamics. The replacement of Ser106 in Arabidopsis with Thr120 in quinoa, although maintaining polarity, could alter the geometry of the interaction site with Lys254 and reduce local stability. Similarly, the substitution of Thr157 with Ala171 eliminated a hydroxyl group, modifying the orientation of Glu172 and disrupting two hydrogen bonds present in AtUVR8. These variations may influence the stability or the kinetics of CqUVR8 re-dimerization. Overall, the models suggest that CqRCC1_20 and CqRCC1_23 preserve a functional homodimeric assembly, stabilized by 28 intermolecular hydrogen bonds, consistent with the organization observed in Arabidopsis UVR8. Nonetheless, differences such as the loss of a β-sheet or specific point substitutions may modulate homodimer stability and the duration of UV-B signaling, indicating structural adaptations of quinoa to high-radiation environments.

### 2.7. Predicted Protein–Protein Interactions of CqUVR8

**Figure 7:**
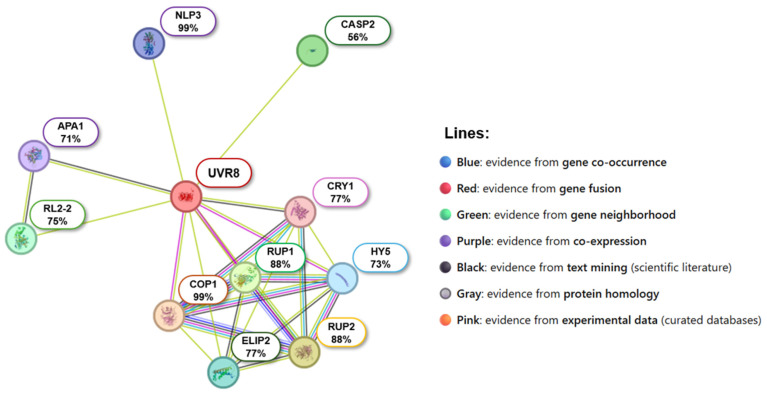
Predicted functional interaction network for UVR8 in *C. quinoa*. The network was generated using the STRING database, based on homology with validated interactions in *A. thaliana*. Nodes represent quinoa homologous proteins, with percentage similarity indicated. Edges of different colors represent the type of evidence supporting functional interactions.

To elucidate potential molecular mechanisms of UVR8 in quinoa, a functional protein interaction network was constructed based on validated *Arabidopsis* interactions (Figure 7). The analysis revealed 10 candidate proteins predicted to directly interact with CqUVR8, identified through sequence similarity and functional prediction. These proteins are implicated in photomorphogenesis regulation, light signaling transduction, and stress adaptation, reflecting a functional architecture parallel to that described in *A. thaliana*. Conserved interactions were identified with COP1, a master regulator of light-dependent development; RUP1 and RUP2, negative modulators of UV-B signaling; and HY5, a key transcription factor in light-induced gene activation. Additional predicted interactions included CRY1, a blue-light photoreceptor, and ELIP2, involved in photoprotection under high irradiance. Furthermore, interactions with CASP2, linked to Casparian strip formation, and APA1, associated with protein turnover, suggest possible integration between UV-B response, structural metabolism, and proteostasis. These predicted associations point to a multifunctional role for UVR8 in quinoa.

### 2.8. Critical Residues in the Photodynamics of CqUVR8

**Figure 8:**
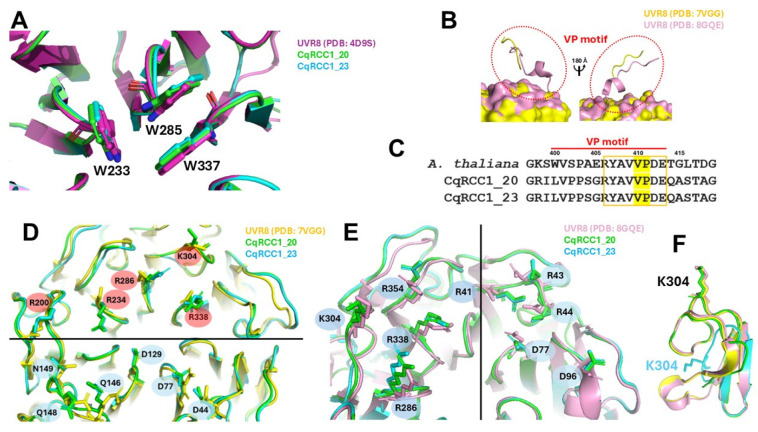
Conserved residues of *C. quinoa* UVR8 involved in interactions with COP1 and RUP2. (**A**) Structural superposition of CqRCC1_20 (green) and CqRCC1_23 (cyan) with *A. thaliana* UVR8 (purple). (**B**) Structural superposition highlighting the VP motif (red circles) in UVR8 from *A. thaliana*, based on UVR8 structures in complex with COP1 and RUP2. The model reflects the conformations adopted by UVR8 during its interaction with COP1 (yellow) and RUP2 (pink). (**C**) Sequence alignment showing VP motif conservation across species. The yellow box highlights residues that are fully conserved among all sequences. (**D**) Conserved UVR8 residues involved in COP1 interaction (highlighted in red and cyan circles). (**E**) Conserved UVR8 residues involved in RUP2 interaction. (**F**) Conservation of residue K304; however, in the CqRCC1_23 model, this residue is not conserved in the predicted 3D structure.

Conservation of key residues involved in UVR8 photodynamic was assessed by superimposing structural models of CqRCC1_20 and CqRCC1_23 with the *A. thaliana* structure (PDB: 4D9S). The tryptophan residues W233, W285, and W337—responsible for UV-B absorption and dimer disruption—were conserved and maintained their three-dimensional orientation in both models (Figure 5A and Figure 8A), suggesting preservation of photoactive mechanisms. The first interaction interface of UVR8 with COP1 and RUP2, proteins associated with UVR8 monomerization and dimerization, respectively, was analyzed through the VP motif, which is essential for recognition by these regulators. In quinoa, point substitutions were detected (W400→L, P402→S, A408→S, E409→G); however, the central residues V410 and P411 remained conserved in both isoforms (Figure 8B,C), suggesting that these variations may modulate interaction affinity without abolishing it.

Electrostatic charges determined the second interaction interface. Comparison of the basic (R338, R286, R234, R200) and acidic (D44, D77, D129, Q148, N149, Q146) residues between Arabidopsis and quinoa UVR8 revealed a conserved spatial arrangement, compatible with binding to the complementary electrostatic surface of COP1 (Figure 8D). Similarly, residues R43, D44, D77, D96, R41, R286, R338, and R354, previously implicated in RUP binding, were conserved in orientation and position (Figure 8E), supporting the maintenance of functional interactions with RUP2. Residue K304, associated with interaction with both regulators, was preserved in the sequence; however, only isoform CqRCC1_20 reproduced a structurally compatible environment in the three-dimensional model. In contrast, the deletion of 24 residues in CqRCC1_23, located in an adjacent region, altered its spatial positioning (Figure 8F), potentially compromising interactions with the regulators. This distinction suggests a functional divergence between quinoa UVR8 isoforms.

## 3. Discussion

This study represents the first comprehensive in silico analysis of the *RCC1* gene family in *Chenopodium quinoa*, with particular focus on one of its members, the UVR8 protein, which is recognized for its role as a specific photoreceptor of UV-B radiation in *Arabidopsis thaliana*. While this signaling pathway has been well characterized in other model species, in quinoa—a crop highly adapted to environments with intense radiation, such as the Andes at altitudes up to 4500 m—the function of the RCC1 family, and especially the *UVR8* gene, had not yet been explored. The characterization presented here provides a crucial foundation for understanding the molecular adaptive mechanisms to UV-B radiation in this pseudocereal.

### 3.1. Identification and Diversity of RCC1 Genes in Chenopodium quinoa

We identified 40 genes containing the *RCC1* domain in the quinoa genome, revealing considerable structural diversity among them. The observed variation in coding sequence (CDS) length, molecular weight, isoelectric point (pI), and aliphatic index is likely associated with the species’ allopolyploid nature. As proposed by Ramsey and Ramsey [15] and Adams [16], chromosomal rearrangements triggered by polyploidy can drive gene family expansions. This may explain the increased gene copy number compared to ancestral progenitors [17], with direct implications for the regulation and functional specialization of duplicated genes. Such processes may influence *RCC1* gene expression levels, as well as promote subfunctionalization or neofunctionalization.

### 3.2. Physicochemical Properties and Subcellular Localization

The pI analysis suggests functional differentiation within the RCC1 family. For example, CqRCC1_15 (pI 4.73) may be adapted to acidic compartments, whereas CqRCC1_40 (pI 9.32) may function in alkaline environments. Although the aliphatic index has been associated with thermal stability in other systems [18], work in *A. thaliana* by Volkening et al. [19] indicates that this relationship may not be straightforward for RCC1 proteins, warranting experimental validation. Regarding subcellular localization, predictions indicate distribution across the nucleus, cytoplasm, mitochondria, and peroxisomes, reinforcing the hypothesis of multifunctionality within the RCC1 family. The predicted mitochondrial localization of CqRCC1_28 suggests a putative functional homolog of RUG3, the only mitochondrial RCC1 member described in *A. thaliana* [20].

### 3.3. Phylogenetic Relationship and Structural Conservation with UVR8

Phylogenetically, quinoa RCC1 members grouped into five clades, showing substantial structural diversification. In *Arabidopsis thaliana*, the RCC1 family comprises 27 members, several of which have diversified into specialized photoreceptors such as UVR8. This provides a useful comparative framework for interpreting the evolutionary expansion observed in quinoa. Of particular interest are CqRCC1_20 and CqRCC1_23, clustered in clade II, due to their high similarity to *A. thaliana* UVR8, a specialized UV-B photoreceptor [21]. Sequence alignments revealed that CqRCC1_20 retains seven β-propeller blades, whereas CqRCC1_23 contains only six, with key tryptophan residues conserved except for W402, replaced by leucine. Notably, this substitution mirrors that reported in *Chlamydomonas reinhardtii* UVR8, which retains photoreceptor functionality despite the change [13,22].

### 3.4. Cis-Regulatory Elements (CREs)

The analysis of cis-regulatory elements complements and strengthens structural evidence supporting CqRCC1_20 and CqRCC1_23 as functional UVR8 homologs. The presence of light-responsive motifs such as GT1-motif, Box 4, and I-box in their promoters suggests gene regulation highly sensitive to light, particularly UV-B. Additionally, CqRCC1_23 contained flavonoid biosynthesis–associated elements (chs-CMA1a), reinforcing its potential role in photoprotection. This transcriptional sensitivity is consistent with the conservation of key residues for UV-B perception (W285, W233, W337) and functional dimerization described in AtUVR8.

The co-occurrence of ABA-responsive (ABRE) and drought-related (MBS) elements suggests that these isoforms may participate in cross-talk between UV-B signaling and other abiotic stress pathways, as reported in other plant systems [23]. This dual layer of structural and transcriptional regulation supports the notion that quinoa has evolved multifactorial strategies to thrive under high-radiation conditions, with isoforms such as CqRCC1_23 potentially conferring an adaptive advantage through faster or more sustained UVR8 activation.

### 3.5. Structural and Functional Conservation of CqUVR8 Models

Predictive models of CqRCC1_20 and CqRCC1_23 revealed a high degree of structural conservation compared with UVR8 photoreceptors from *A. thaliana* [13,14], *Malus domestica* [24], *Populus euphratica* [25], and *Chrysanthemum morifolium* [26]. CqRCC1_20 retained the canonical seven-bladed β-propeller fold, whereas CqRCC1_23 displayed only six blades due to a structural deletion spanning residues F318–Q334 (Figure 3). This absence, together with additional substitutions, may compromise the stability of the central domain, suggesting a fine-tuned adaptive adjustment. Both models conserve GWRHT motifs in blades 5, 6, and 7, which are essential for UV-B perception. The preservation of W233, W285, and W337 supports functional photoreceptor competence, consistent with structural studies in *A. thaliana* [13,14]. Complementary electrostatic surfaces were identified between subunits, forming 28 intermolecular hydrogen bonds between spatially conserved charged residues, similar to those reported in *P. euphratica* [25] and *M. domestica* [24], thereby supporting stable homodimerization.

To date, structural studies of UVR8 in plant species other than *Arabidopsis thaliana* have relied on general homology-based modeling approaches with limited structural resolution [24,25,26]. The only experimentally solved UVR8 structure available corresponds to *A. thaliana* [13,14]. Superposition of this structure with the models of CqRCC1_20 and CqRCC1_23 yielded RMSD values of 0.326 Å and 0.409 Å, respectively, confirming the high structural similarity between *A. thaliana* UVR8 and the *Chenopodium quinoa* homologs. Comparative analysis identified the substitutions Ser106→Thr120 and Thr157→Ala171 in CqUVR8, which weaken the hydrogen-bond network at the dimer interface (Figure 6). The first substitution alters the interaction with Lys254, whereas the second removes a hydroxyl group required for stabilizing Glu158, resulting in the loss of two hydrogen bonds. Additionally, Arg328 in CqRCC1_23 does not participate in salt-bridge formation as observed in AtUVR8. The *C. quinoa* model predicts 28 intermolecular hydrogen bonds, compared to 36 in AtUVR8 [13,14], suggesting reduced dimer stability and a higher propensity for monomerization—an active state associated with enhanced UV-B signaling [27].

Comparable functional and structural features have also been documented in major crops such as maize (*Zea mays*) and rice (*Oryza sativa*). In maize, ZmUVR8 shares approximately 73% identity with AtUVR8 and conserves the key tryptophan residues required for UV-B perception [28]. Similarly, the rice homologs OsUVR8a and OsUVR8b retain the core seven-bladed β-propeller architecture and preserve the essential residues involved in UV-B sensing, as supported by homology-based structural models [29]. Together, these findings indicate that the UV-B receptor framework is highly conserved across diverse crop lineages. When integrated with our structural analysis of CqUVR8, this broader evidence suggests that the specific molecular adjustments observed in quinoa—particularly those associated with increased monomerization propensity—may reflect adaptive strategies that enhance its resilience to the intense UV-B radiation characteristic of Andean environments.

The interactions described in the functional network of CqUVR8 were inferred through homology-based prediction, using validated interactions from *Arabidopsis thaliana* as reference. Although the analysis suggests a conserved architecture in light signaling and UV-B stress response, these findings remain speculative until experimentally confirmed. The identification of candidate proteins such as COP1, RUP2, HY5, CRY1, ELIP2, CASP2, and APA1 supports relevant functional hypotheses; however, their direct interaction with CqUVR8 requires validation through experimental approaches, including heterologous co-expression, co-immunoprecipitation assays, protein–protein interaction analyses, and UV-B-induced expression studies. Confirming these associations will help clarify the multifunctional role of UVR8 in *Chenopodium quinoa* and its contribution to adaptation in high-radiation environments.

Following this analysis, the regulatory proteins COP1 and RUP2—responsible for UVR8 monomerization and redimerization, respectively—were selected for further evaluation. A partial conservation of CqUVR8 residues involved in interactions with COP1 and RUP was identified. Within the VP motif (Figure 8B,C), which is critical for recognition by both regulators, several substitutions were detected (W400L, S402P, A408S, E409G), while residues V410 and P411 remained conserved. The preservation of these latter residues suggests a retained interaction capacity with COP1 and RUP2, consistent with observations in other species [30,31]. However, the remaining substitutions may alter the relative binding affinity of CqUVR8 toward COP1 and RUP2, indicating a potentially distinct functional behavior, possibly favoring a predominantly monomeric state or an enhanced redimerization capacity.

Finally, in the structural analysis, in addition to the VP motif, other key residues were identified in CqUVR8 that enable electrostatic interactions with COP1 and RUP (Figure 8D–F). These residues are conserved in both CqRCC1_20 and CqRCC1_23. However, the deletion of 24 residues in CqRCC1_23 alters the structural conformation of K304, preventing its interaction with COP1 and RUP and consequently affecting the oligomeric state of CqUVR8. This observation supports the existence of functionally divergent UVR8 isoforms in quinoa, potentially arising from gene duplication or polyploidy events [32,33,34].

Collectively, the substitutions affecting the hydrogen bond network at the homodimeric interface, the modifications within the VP motif, and the structural deletion that disrupts COP1 and RUP interaction sites in CqRCC1_23 indicate an altered monomer–dimer dynamic in CqUVR8. This structural variation suggests a molecular adaptation associated with sustained UV-B response in high-radiation environments, such as the Andean ecosystems where *C. quinoa* thrives. The observed reduction in hydrogen bonding supports the hypothesis that CqUVR8 adopts a predominantly monomeric conformation, a state that triggers signaling pathways linked to recovery from radiation-induced stress. These findings open new avenues for functional assays aimed at validating the differential behavior of UVR8 isoforms and their role in ultraviolet radiation tolerance.

## 4. Materials and Methods

### 4.1. Identification of RCC1 Family Members in the Chenopodium quinoa Genome

RCC1 proteins in *Chenopodium quinoa* Willd. were identified using the RCC1 sequence from *Arabidopsis thaliana*, retrieved from The Arabidopsis Information Resource (TAIR; release 10, https://www.arabidopsis.org/, accessed on 15 January 2025) as the reference query. BLASTP searches were performed against the *C. quinoa* genome (v1.0) in Phytozome v13 (https://phytozome-next.jgi.doe.gov/, accessed on 17 January 2025) using an E-value threshold of 1 × 10^−20^, complemented with keyword searches using “RCC1.”

Candidate sequences were validated through domain inspection using Pfam v38.0 (http://pfam.xfam.org/, accessed on 19 January 2025), NCBI Conserved Domain Database (CDD) v3.19 (https://www.ncbi.nlm.nih.gov/cdd, accessed on 20 January 2025), and InterPro release 97.0 (https://www.ebi.ac.uk/interpro/, accessed on 20 January 2025) to confirm the presence of conserved RCC1 domains. Only sequences containing at least one canonical RCC1 repeat were retained, and validated genes were designated with the prefix “CqRCC1” followed by a sequential number.

Physicochemical properties—including amino acid length, molecular weight, isoelectric point, and aliphatic index—were computed using ProtParam (ExPASy; release 3.0, https://expasy.org/, accessed on 21 January 2025). Subcellular localization was predicted using CELLO v2.5 (http://cello.life.nctu.edu.tw, accessed on 21 January 2025), PSORT II server (version 2.0, https://genscript.com/psort.html, accessed on 21 January 2025), and TargetP v2.0 (https://services.healthtech.dtu.dk/services/TargetP-2.0/, accessed on 21 January 2025).

### 4.2. Sequence Alignment and Phylogenetic Reconstruction

Multiple sequence alignment was performed with the 40 RCC1 proteins from *C. quinoa* and 27 sequences from *A. thaliana*, using T-Coffee v13.45.0.4846268 software (https://www.ebi.ac.uk/jdispatcher/msa/tcoffee, accessed on 10 March 2025) with the BLOSUM62 substitution matrix, and the alignments were visualized in JALVIEW v2.11.4.1. Phylogenetic reconstruction was performed in IQ-TREE v2.3.5 (http://iqtree.cibiv.univie.ac.at/, accessed on 10 March 2025). The phylogenetic tree reconstruction was performed using the maximum likelihood inference method with a Jones-Taylor-Thornton (JTT + F + I + 4G) model. Node support was evaluated using 1000 bootstrap replicates. The tree topology was extracted in Newick format for visualization and editing in iTOL v7.2.2 (https://itol.embl.de, accessed on 12 March 2025).

### 4.3. Gene Structure and Motif Analysis

Genomic DNA and CCDS sequences were obtained from the *CqRCC1* genes of the *C. quinoa* genome, deposited in the Phytozome v13 database (https://phytozome-next.jgi.doe.gov/, accessed 21 March 2025). Exon and intron structures were predicted using the Gene Structure Display Server v2.0 (GSDS) (https://gsds.gao-lab.org/, accessed on 20 April 2025) and edited using TBtools v2.019 software based on genomic coordinates. Conserved domains were annotated using the NCBI conserved domains function with the CDD v3.21 data search function with default parameters (https://www.ncbi.nlm.nih.gov/Structure/wrpsb-out/wrpsb.cgi, accessed on 20 April 2025) and EMBL InterPro (https://www.ebi.ac.uk/interpro/, accessed on 20 April 2025), while conserved motifs were identified using MEME Suite v5.5.8 (https://meme-suite.org/, accessed on 22 April 2025), specifying up to 10 motifs per protein with default parameters.

### 4.4. Structural Modeling of CqUVR8 Proteins

Three-dimensional models of CqRCC1_20 and CqRCC1_23 were generated using homology modeling in SWISS-MODEL from Expasy (https://swissmodel.expasy.org/interactive, accessed on 30 April 2025), using the AtUVR8 crystal structure (PDB: 4D9S) as a template. The models were visualized and analyzed in PyMOL v2.4.5 (Schrödinger). Structural quality was assessed using GMQE and QMEAN scores and validated using Ramachandran plots in Procheck.

### 4.5. Prediction of Protein Interactions

Potential interactions of *C. quinoa* UVR8 were inferred from the known interactome of AtUVR8 (AT5G63860) in *A. thaliana*, as retrieved from STRING v12.0 (https://string-db.org/, accessed on 5 May 2025). Candidate interactors were validated by BLASTP (https://blast.ncbi.nlm.nih.gov/Blast.cgi?PAGE=Proteins, accessed on 5 May 2025) searches in Phytozome v13 (https://phytozome-next.jgi.doe.gov/, accessed on 5 May 2025) and NCBI to confirm their orthologs in *C. quinoa*. Sequence similarity levels are presented in Figure 7.

### 4.6. Analysis of Residues Involved in Photodynamics

To identify residues critical for UVR8 photodynamics, dimeric and monomeric models of CqUVR8 were compared. Conservation of residues reported as essential in AtUVR8 was examined, with emphasis on those implicated in UV-B–induced dissociation. Structural analyses were performed in PyMOL v3.1, evaluating spatial orientation and stabilizing interactions in both conformations.

## 5. Conclusions

The CqUVR8 models were constructed based on the structural characterization of AtUVR8 as a functional homodimer. Although bioinformatic tools enabled detailed identification and analysis of UVR8 homolog candidates in quinoa, several limitations remain: (i) lack of experimental validation through crystallography or cryo-electron microscopy, (ii) absence of transcriptomic data from tissues exposed to UV-B radiation, and (iii) need for functional assays such as heterologous expression or complementation in *Arabidopsis* UVR8 mutants. Future studies should address these aspects to confirm the functional relevance of CqUVR8 isoforms and their role in UV-B radiation tolerance.

## Figures and Tables

**Table 1:** Physicochemical properties and predicted subcellular localization of the 40 RCC1 family proteins identified in *Chenopodium quinoa*.

Gene Name	Transcript ID	Localization	CDS (pb)	AA	Weight (kDa)	pI	Aliphatic Index	Subcellular Location
*CqRCC1_1*	AUR62012141	Scaffold_1995:925914..931012 f	1494	497	52.14	5.76	72.23	Me ^1,2^, C ^1,2^
*CqRCC1_2*	AUR62024206	Scaffold_4119:1567432..1570457 f	1506	501	53.79	6.09	80.4	Me ^1,2^, C ^1,2^
*CqRCC1_3*	AUR62023679	Scaffold_2187:562911..569141 r	1401	466	49.81	5.75	88.91	Cl ^1^, Mi ^2,3^
*CqRCC1_4*	AUR62019141	Scaffold_3876:3492836..3502921 r	1671	556	60.42	6.94	59.41	Ex ^1,2^, N ^1,2^
*CqRCC1_5*	AUR62026458	Scaffold_4244:6102177..6104925 f	750	249	26.64	8.57	84.14	Me ^1^, C ^2^
*CqRCC1_6*	AUR62026456	Scaffold_4244:6090841..6091515 f	483	160	17.38	6.42	81.06	Me ^1^, N ^2^
*CqRCC1_7*	AUR62034255	Scaffold_1337:1246314..1251339 r	1446	481	51.94	6.99	84.28	Me ^1^, C ^1,2^
*CqRCC1_8*	AUR62002142	Scaffold_4480:2651092..2655743 f	3861	1286	139.78	5.61	69.21	Me ^1^, N ^2^
*CqRCC1_9*	AUR62002515	Scaffold_4480:7952067..7961272 f	1176	391	42.09	4.98	84.27	Ex ^1,2^, M ^1,2^
*CqRCC1_10*	AUR62013542	Scaffold_1611:4718188..4727510 r	3402	1133	122.57	8.61	74.63	Me ^1,2^, N ^1,2^
*CqRCC1_11*	AUR62003547	Scaffold_2370:1986325..1990982 r	1254	417	43.33	6.24	85.08	P ^1^, M ^1^
*CqRCC1_12*	AUR62039706	Scaffold_2419:242891..246491 r	1656	551	59.37	4.9	77.66	P ^1,2^, C ^1,2^
*CqRCC1_13*	AUR62039726	Scaffold_2419:720240..730637 f	3162	1053	114.57	9.23	80.85	Me ^1^, N ^2^
*CqRCC1_14*	AUR62020143	Scaffold_2465:1185020..1187510 r	2490	829	90.09	7.65	90.05	Me ^1^, N ^2^
*CqRCC1_15*	AUR62044490	Scaffold_1723:8..5728 r	903	301	33.74	4.73	84.95	C ^1,2^
*CqRCC1_16*	AUR62009274	Scaffold_3674:3391734..3394917 f	1563	520	55.96	4.97	73.85	P ^1,2^, C ^1,2^
*CqRCC1_17*	AUR62009482	Scaffold_3674:5764320..5769103 f	939	312	33.37	6.58	86.57	Ex ^1,2^, N ^1,2^
*CqRCC1_18*	AUR62023161	Scaffold_1189:2007096..2011966 f	1497	498	53.66	6.24	83.03	Me ^1,2^, C ^1,2^
*CqRCC1_19*	AUR62000563	Scaffold_2088:6049517..6064591 r	1161	387	41.43	6.35	79.84	Me ^3^, N ^1^, M ^2^
*CqRCC1_20*	AUR62000528	Scaffold_2088:5597182..5606640 f	1356	451	47.85	5.77	73.1	Ex ^1,2^, M ^1,2^
*CqRCC1_21*	AUR62000180	Scaffold_2088:1942924..1949822 r	3216	1071	115.86	9.09	67.63	Me ^1,2^, N ^1,2^
*CqRCC1_22*	AUR62040694	Scaffold_1465:99228..104321 r	1482	493	51.33	5.47	71.26	Me ^1,2^, C ^1,2^
*CqRCC1_23*	AUR62006875	Scaffold_3429:5939170..5947925 f	1305	434	46.23	6.06	74.15	Ex ^1,2^, M ^1,2^
*CqRCC1_24*	AUR62006916	Scaffold_3429:6412259..6424318 r	1281	426	45.48	6.27	83.26	Ex ^1^, M ^2,3^
*CqRCC1_25*	AUR62006531	Scaffold_3429:2616822..2623416 r	3216	1071	115.96	9.06	67.98	Me ^1,2^, N ^1,2^
*CqRCC1_26*	AUR62029360	Scaffold_2939:2226660..2232351 r	1467	488	52.60	7.24	83.67	Me ^1,2^, C ^1,2^
*CqRCC1_27*	AUR62014530	Scaffold_1566:4165280..4170210 f	1095	364	39.44	6.33	79.29	P ^1,2^, C ^1,2^
*CqRCC1_28*	AUR62014404	Scaffold_1566:2639482..2646019 r	1512	503	54.57	8.94	65.88	Me ^1^, Mi ^2^
*CqRCC1_29*	AUR62028803	Scaffold_2837:713405..734287 f	3204	1067	115.97	8.66	70.36	Me ^1,2^, N ^1,2^
*CqRCC1_30*	AUR62026740	Scaffold_2081:4343447..4353350 f	3402	1133	122.53	8.58	74.97	Me ^1,2^, N ^1,2^
*CqRCC1_31*	AUR62026265	Scaffold_3298:5035900..5041104 f	1083	360	38.23	6	78.56	Ex ^1,2^, N ^1,2^
*CqRCC1_32*	AUR62037183	Scaffold_1237:49943..56961 r	2670	889	95.54	8.91	70.29	Me ^1,2^, N ^1,2^
*CqRCC1_33*	AUR62026876	Scaffold_2185:1349875..1363405 r	3318	1105	119.45	9.05	73.13	Me ^1^, N ^2^
*CqRCC1_34*	AUR62015566	Scaffold_2751:8118037..8123109 r	3888	1295	140.62	5.87	69.05	Me ^1^, N ^2^
*CqRCC1_35*	AUR62008975	Scaffold_1710:8511136..8515728 r	1125	374	40.62	6.52	80.8	P ^1,2^, N ^1,2^
*CqRCC1_36*	AUR62038079	Scaffold_1613:1603591..1604910 f	444	147	16.11	6.94	108.03	C ^1,2^, Ex ^1,2^
*CqRCC1_37*	AUR62005755	Scaffold_1214:7543923..7557818 r	3096	1031	112.25	8.41	69.7	Me ^1,2^, N ^1,2^
*CqRCC1_38*	AUR62005531	Scaffold_1214:4144473..4148294 r	1506	501	53.78	6.2	79.82	Me ^1,2^, N ^1,2^
*CqRCC1_39*	AUR62017861	Scaffold_3086:1094434..1100940 r	993	330	34.44	6.45	82.12	Me ^1^, Re ^2^
*CqRCC1_40*	AUR62036278	Scaffold_2738:983326..992737 f	3081	1026	111.84	9.32	82.11	Me ^1^, N ^2^

Abbreviations: (ID): Identifiers, (r) reverse, (f) forward, (CDS) coding sequence length, (AA) number of amino acids, (kDa) molecular weight and (pI) isoelectric point. Subcellular localization was predicted using ^1^ CELLO, ^2^ PSORT, and ^3^ Target P servers. The following abbreviations are defined for localization: (Me) Endoplasmic Membrane, (C) Cytoplasm, (Cl) Chloroplast, (Re) Endoplasmic Reticulum, (N) Nucleus, (Ex) Extracellular Membrane, (Mi) Mitochondrion, (M) Membrane, and (P) Peroxisome.

## Data Availability

The data presented in this study are available on request from the corresponding author.

## Supplementary Materials

The following supporting information can be downloaded at: https://www.mdpi.com/article/10.3390/ijms262311657/s1.
