# Supplementary material for: In Silico Characterization of the RCC1 Family and the UVR8 Gene in Chenopodium quinoa Willd
Source: Int J Mol Sci. 2025 Dec 1;26(23):11657. doi: 10.3390/ijms262311657 (PMC12692308; doi:10.3390/ijms262311657)
Supplement: Supplementary file 1 [file ijms-26-11657-s001.zip › ijms-3959977-supplementary.pdf]

Supplementary Figure S1: CLUSTAL W CqRCC1 multiple sequence alignment

```
CqRCC1_1     MNGGEGE---GGESEMQMEVEMGEEERERRVYMWGYLPGALPQRT-----
CqRCC1_10    MADLVS----YGNADRDIEQA--LIALKKGAQLLKYGRKGKPKFYPFRLS
CqRCC1_11    KPPLLY--------------------------------------------
CqRCC1_12    MADVTE----TPE-------------------------------------
CqRCC1_13    MEGSLSP---RGHKQS-------------------------PQ-------
CqRCC1_14    MKKKAI----YFSLVR----------------------RC--QLLPVL--
CqRCC1_15    MVEMAT----ESRED--------------SVYMAKLAEQAE---------
CqRCC1_16    MADVTE----SPG-------------------------------------
CqRCC1_17    MWGQNQ----GSNY------------------------------------
CqRCC1_18    MEIGEI----LGES------------------------------------
CqRCC1_19    MWTRAV----YSQ-------------------------------------
CqRCC1_2     MDATTS----GTETI--------------------QY-------------
CqRCC1_20    MGTIEK----D---------------------------------------
CqRCC1_21    MDKMTTDLTRNGPVERDIEQA--ITALKKGAYLLKYGRRGKPKFCPFRLS
CqRCC1_22    MNGGEGE---GGGSEMQMEVEMGEEERERRVYMWGYLPGALPQRT-----
CqRCC1_23    MGTIEK----D---------------------------------------
CqRCC1_24    MWTRAV----YSQ-------------------------------------
CqRCC1_25    MDKMTTDLTRNGPVERDIEQA--ITALKKGAYLLKYGRRGKPKFCPFRLS
CqRCC1_26    MAVSID----ELPSH-----------------------------------
CqRCC1_27    MPSDT---------------------------------------------
CqRCC1_28    MAEVEV----EKKC------------------------------------
CqRCC1_29    MGDSTR----GGPGERDIEQA--VTALKKGATLLKYGRRGKPKFCPFRLS
CqRCC1_3     MAHNLKL----NEMM-----------------------------------
CqRCC1_30    MADLVS----YGNADRDIEQT--LIALKKGAQLLKYGRKGKPKFCPFRLS
CqRCC1_31    MEEQEK----AFKKQKEIN-------------------------------
CqRCC1_32    MDWIPFI-------------------------------------------
CqRCC1_33    MNADQAR---AGPVERDIELA--ITALKKGTNLLKYGRRGKPKFCPFRLS
CqRCC1_34    MWRRN------------EYEV--ICSLKNHQ----------------NYC
CqRCC1_35    MLMKL---------------------------------------------
```

```
CqRCC1_36     MHYASF----S---------------------------------------
CqRCC1_37     MGDSTR----GGPGERDIEQA--VTALKKGATLLKYGRRGKPKFCPFRLS
CqRCC1_38     MDATTS----GTETI-------------------QY--------------
CqRCC1_39     MLK-----------------------------------------------
CqRCC1_4      MAEVEV----EKKC------------------------------------
CqRCC1_40     MECSLSP---RGHKQS-------------------------PQ-------
CqRCC1_5      ML------------------------------------------------
CqRCC1_6      MEEQEK----AVKKQKEIN-------------------------------
CqRCC1_7      MAVSID----ELPSH-----------------------------------
CqRCC1_8      DE------------------------------------------------
CqRCC1_9      DDRSGESAEDVGNSD-----------------------------------


CqRCC1_1      --------------------------------------------------
CqRCC1_10     SDETTLIWLSSGGEKS---------------------------------I
CqRCC1_11     --------------------------------------------------
CqRCC1_12     --------------------------------------------------
CqRCC1_13     ---------SHG-RKS----------------------------------
CqRCC1_14     --LTLFFLLNCTIVRG---------------------------------L
CqRCC1_15     --------------------------------------------------
CqRCC1_16     --------------------------------------------------
CqRCC1_17     --------------------------------------------------
CqRCC1_18     --------------------------------------------------
CqRCC1_19     ---------------F---------------------------------S
CqRCC1_2      ---------CNIPEQPIIGSLVTSPRPAFLRQVTCSFFRHPANFAPDYNL
CqRCC1_20     --QTAVA-------------------------------------------
CqRCC1_21     NDESVLIWFSGKEEKH---------------------------------L
CqRCC1_22     --------------------------------------------------
CqRCC1_23     --QTAVV-------------------------------------------
CqRCC1_24     ---------------I---------------------------------S
CqRCC1_25     NDESVLIWFSGKEEKH---------------------------------L
```

CqRCC1_26 ----------------IVLEILTSGRL--------------------SAA

CqRCC1_27 --

CqRCC1_28 --

CqRCC1_29 NDESVLIWFSGKEEKQ---------------------------------L

CqRCC1_3 --

CqRCC1_30 SDETTLIWLSSGGEKS---------------------------------I

CqRCC1_31 -----------EEEQQ----------------------------------

CqRCC1_32 ------------PTRV---------------------------------L

CqRCC1_33 NDESVLVWYSGNEEKH---------------------------------L

CqRCC1_34 SDETTLIWLSHGEERN---------------------------------L

CqRCC1_35 --

CqRCC1_36 --C---

CqRCC1_37 NDESVLIWYSGKEEKQ---------------------------------L

CqRCC1_38 ---------CNIPEQPIVGSLVTSPRPAFLRQVTCSFFRHPANFAPDYDL

CqRCC1_39 --

CqRCC1_4 --

CqRCC1_40 ---------SHG-RKS----------------------------------

CqRCC1_5 --

CqRCC1_6 -----------EEEQQ----------------------------------

CqRCC1_7 ----------------IVLEILTSGRL--------------------SAA

CqRCC1_8 ---TTLIWLSHGEERN---------------------------------L

CqRCC1_9 --

CqRCC1_1 --

CqRCC1_10 K---LSCVSKVIPGQRTA---------------VFRRYLRPDKDYLSFSL

CqRCC1_11 --

CqRCC1_12 --

CqRCC1_13 ----------V---------------------------------------

CqRCC1_14 G---SSSTIAAIHGFNT---------------------------------

CqRCC1_15 ------------R-------------------------Y-----------

CqRCC1_16 --

CqRCC1_17 --

CqRCC1_18 --------KPVSV-------------------------------------

CqRCC1_19 K---SSILN---PNL-----------------------------------

CqRCC1_2 S---IAELAAHDMCQKRA---------------IFRPMTPEQKE------

CqRCC1_20 --------AAAAKEDRS---------------------------------

CqRCC1_21 K---LSHVSRIISGQRT---------------------------------

CqRCC1_22 --

CqRCC1_23 --------SAVAKEDRS---------------------------------

CqRCC1_24 K---LSILN---PNL-------------------------------GLGL

CqRCC1_25 K---LSHVSRIISGQRT---------------------------------

CqRCC1_26 DLVCLELASSI-FGGSQG---------------LYPLKFRSLADLAAFQL

CqRCC1_27 --

CqRCC1_28 --------EEEKV-------------------------------------

CqRCC1_29 K---LSQVSRIIPGQRT---------------GIFKRYPRPEKEYQSFSL

CqRCC1_3 --

CqRCC1_30 K---LSCVSKVIPGQRTA---------------VFRRFLRPDKDYLSFSL

CqRCC1_31 ---------------E----------------------------------

CqRCC1_32 K---

CqRCC1_33 K---LSHVSRIIPGQRTIISGLVVLVNLKVMQPIFQRYPRPEKEYQSFSL

CqRCC1_34 K---LSVVSKIIQGQRTA---------------VFRRFLRPEKDYLSFSL

CqRCC1_35 --

CqRCC1_36 --

CqRCC1_37 K---LSQVSRIIPGQRT---------------GIFKRYPRPEKEYQSFSL

CqRCC1_38 S---IAELAAHDMCQKRA---------------IFRPMTPEQKE------

CqRCC1_39 --

CqRCC1_4 --------EEEKV-------------------------------------

CqRCC1_40 ----------V---------------------------------------

CqRCC1_5 --

CqRCC1_6 ---------------E----------------------------------

CqRCC1_7 DLVCLELASTI-FGGSQG---------------LYPLKFRSLADLAAFQL

CqRCC1_8 K---LSVVSKIIQGQRTA---------------VFRRFLRPEKDYLSFSL
CqRCC1_9 --

CqRCC1_1 --
CqRCC1_10 IYNNGKRSLDLICKE------------KVEAEVWIAGLTALVSAGKG---
CqRCC1_11 --
CqRCC1_12 --
CqRCC1_13 -FGVTQKDIWLIVKE------------GCLADVELA-LTQLKK-NGGNIN
CqRCC1_14 -----------VCGILVGKSQGIQCFQQGRV------FSALPN-------
CqRCC1_15 ----------------------------EE-----------------M--
CqRCC1_16 --
CqRCC1_17 -----------------------------P--------------------
CqRCC1_18 -------------------------PTKSA--------------------
CqRCC1_19 GIGFCSRLYSTVERG------------KRF--------------------
CqRCC1_2 ---------------------------------------DLKQRCGG---
CqRCC1_20 ---------------------GFTP-----------------Q-------
CqRCC1_21 -----------ICKD------------KDEAEVWFSGLKALISRSQQ---
CqRCC1_22 --
CqRCC1_23 ---------------------GFTP-----------------Q-------
CqRCC1_24 GLGFCSRLYSTVERG------------KRF--------------------
CqRCC1_25 -----------ICKD------------KDEAEVWFSGLKALISRTQQ---
CqRCC1_26 CVSHSV-----------------------YAPMSFETRKELFDRCGG---
CqRCC1_27 --
CqRCC1_28 --------------------------KGGE--------------------
CqRCC1_29 IYND-SRSLDLICKD------------KDEAEVWFVGLKALISKNGW---
CqRCC1_3 ------------------------------Q---F-----L---------
CqRCC1_30 IYNNRKRSLDLICKE------------KVEAEVWIAGLTALVSAGKG---
CqRCC1_31 ------------------------------REI-----------------
CqRCC1_32 --
CqRCC1_33 IYSD--RSLDVICKD------------KDEAEVWFTGLKALISRYHR---

CqRCC1_34 IYNHGDRSLDVICKD------------KAEAEVWLAGLKALIGLQRN---

CqRCC1_35 --

CqRCC1_36 --

CqRCC1_37 IYND-SRSLDLICKD------------KDEAEVWFVGLKALISKNGW---

CqRCC1_38 ---------------------------------------DLKQRCGG---

CqRCC1_39 --

CqRCC1_4 --------------------------KGGE--------------------

CqRCC1_40 -LGGTQKDIWLIVKE------------GCLADVELA-LTQLKK-NGGNIN

CqRCC1_5 --

CqRCC1_6 ------------------------------REN-----------------

CqRCC1_7 CVSHSV-----------------------YAPMSFETRKELFDRCGG---

CqRCC1_8 IYNHGDRSLDVICKD------------KVEAEVWLAGLKALIGQQRN---

CqRCC1_9 ------------------------------EDSRG-----V---------

CqRCC1_1 --

CqRCC1_10 G------------RSKIDGW--NDEGLSLQ----------DNKDL-----

CqRCC1_11 -------KPTNPSNDAV---------------------------------

CqRCC1_12 --

CqRCC1_13 SRNAFGLTPLHIATWRNH--------------------------------

CqRCC1_14 --

CqRCC1_15 --

CqRCC1_16 --

CqRCC1_17 --

CqRCC1_18 --

CqRCC1_19 --

CqRCC1_2 -------------SWKLV--------------------------------

CqRCC1_20 ----------T---------------------------------------

CqRCC1_21 R------------KWRTESRSD---SIPSEANSPRTYTRRSSPLHSPFAS

CqRCC1_22 --

CqRCC1_23 ----------T---------------------------------------

CqRCC1_24 --

CqRCC1_25 R------------KWRTESRSD---SIPSEANSPRTYTRRSSPLHSPFAS

CqRCC1_26 -------------NWKRV--------------------------------

CqRCC1_27 --

CqRCC1_28 --

CqRCC1_29 R------------KWKNEARES---NTSSADGSTN------RRIHQTIAS

CqRCC1_3 --------------WKRS--------------------------------

CqRCC1_30 G------------RSKIDGW--NDEGLSLQ----------DNKDL-----

CqRCC1_31 --

CqRCC1_32 --

CqRCC1_33 -------------RPRVDPRS----GLSSEANSPKAQTQRSSPLSSPFGS

CqRCC1_34 R------------RTRSEISDLNEGEYSQH----------GRPVL-----

CqRCC1_35 --

CqRCC1_36 --

CqRCC1_37 R------------KWKNEARES---STSSADGSTN------RRIHQSIAS

CqRCC1_38 -------------SWKLV--------------------------------

CqRCC1_39 --

CqRCC1_4 --

CqRCC1_40 LRNAFGLTPLHIATWRNH--------------------------------

CqRCC1_5 --

CqRCC1_6 --

CqRCC1_7 -------------NWKRV--------------------------------

CqRCC1_8 R------------RTRSEISDLNEGEYSQH----------GRPVL-----

CqRCC1_9 --------------SQEE--------------------------------

CqRCC1_1 ----------------PLQS--------------------------PTIV

CqRCC1_10 ------------TSSSPSDSSLTLTRYLSSPEDSFSFHGGFSPKHNQS--

CqRCC1_11 --

CqRCC1_12 --

CqRCC1_13 --

CqRCC1_14 --

CqRCC1_15 --

CqRCC1_16 --

CqRCC1_17 --

CqRCC1_18 --

CqRCC1_19 --

CqRCC1_2 --

CqRCC1_20 --

CqRCC1_21 GDSLQKDSSEHLRLHSPYDS--------------------------PP--

CqRCC1_22 ----------------PLQS--------------------------PIIV

CqRCC1_23 --

CqRCC1_24 --

CqRCC1_25 GDSLQKDSSEHLRLHSPYDS--------------------------PP--

CqRCC1_26 --

CqRCC1_27 --

CqRCC1_28 --

CqRCC1_29 HDAG------DAPCQVPSES--------------------------PP--

CqRCC1_3 --

CqRCC1_30 ------------TSSSPSDSSLTLTRYLSSPEDSFSFHGGFSPKHNQS--

CqRCC1_31 --

CqRCC1_32 --

CqRCC1_33 GDGSQKDGLDP---LYPYES--------------------------PQ--

CqRCC1_34 ------------EAASSVRS--------------------------RP--

CqRCC1_35 --

CqRCC1_36 --

CqRCC1_37 HDAG------DAPCQVPIES--------------------------PP--

CqRCC1_38 --

CqRCC1_39 --

CqRCC1_4 --

CqRCC1_40 --

CqRCC1_5 --

```
CqRCC1_6     --------------------------------------------------
CqRCC1_7     --------------------------------------------------
CqRCC1_8     ------------EAASSVRS--------------------------RP--
CqRCC1_9     --------------------------------------------------


CqRCC1_1     KLPPS--IGTFW--------------------------------------
CqRCC1_10    ----GNFVSSEKSHV--------------------------A-SENT---
CqRCC1_11    --------------------------------------------------
CqRCC1_12    --------------------------------------------------
CqRCC1_13    ----------------------------------------------IPIV
CqRCC1_14    ------------ISYE----------------------------------
CqRCC1_15    --------------------------------------------------
CqRCC1_16    --------------------------------------------------
CqRCC1_17    --------------------------------------------------
CqRCC1_18    --------------------------------------------------
CqRCC1_19    --------------------------------------------------
CqRCC1_2     ---------------LRYLLAG-EACCR-----------------RE---
CqRCC1_20    -----------LRQIL----------------------------------
CqRCC1_21    ----KNALDKAVSDVML-TAPPKGFFPSDSAAMSVHSIS-SG-SDSV---
CqRCC1_22    KLPPS--IGTFW--------------------------------------
CqRCC1_23    -----------LRQIL----------------------------------
CqRCC1_24    --------------------------------------------------
CqRCC1_25    ----KNALDKAVSDVML-TAPPKGFFPSDSAAMSVHSIS-SG-SDSV---
CqRCC1_26    ---------------LRFLQSV-EQASD------IVKTS-----AGN---
CqRCC1_27    --------------------------------------------------
CqRCC1_28    --------------------------------------------------
CqRCC1_29    ----QTGLGKAFSDIILYTAAS-NNCSQTEASTYVASFPPSNSVE-----
CqRCC1_3     ----------------------------------------------V---
CqRCC1_30    ----GNFMSSEKSHV--------------------------A-SENT---
CqRCC1_31    --------------------------------------------------
```

CqRCC1_32 ----KNGLETALSDAILYTVPAKSFMPPESASSSVHSFS-SGGFDGL---

CqRCC1_33 ----KNGLETALSDAILYTVPAKSFIPPESASSSVHSFS-SGGFDGL---

CqRCC1_34 ----SLDLGPLSSDV--------------------------G-SEQA---

CqRCC1_35 --

CqRCC1_36 --

CqRCC1_37 ----QTGLGKAFSDIILYTAAS-NNCSQAEASIYVASFPPSNSVE-----

CqRCC1_38 ---------------LRYLLAG-EACCR-----------------RE---

CqRCC1_39 --

CqRCC1_4 --

CqRCC1_40 --IPIV

CqRCC1_5 --

CqRCC1_6 --

CqRCC1_7 ---------------LRFLQSV-EQASD------------------I---

CqRCC1_8 ----SLDLGPLSSDV--------------------------G-SEQA---

CqRCC1_9 --V---

CqRCC1_1 --KDVC-------GGGCGFAMAI----------------SDTG-------

CqRCC1_10 NMQAKG-------SASDVFRVSVSSAPSTSSHGSAGDDCDALG-------

CqRCC1_11 --

CqRCC1_12 --

CqRCC1_13 RRLLAA-------GADPDARDG-------------ESGWSNLHRALYFGH

CqRCC1_14 --AISG-------GRDFF--CAL----------------SSGGS------

CqRCC1_15 --

CqRCC1_16 --

CqRCC1_17 --

CqRCC1_18 --

CqRCC1_19 --

CqRCC1_2 KSQAIA-------GPGHS--IAV----------------TSTG-------

CqRCC1_20 --LVSA-------GASHS--VAL----------------LSGN-------

CqRCC1_21 YSQMKG-------MGADTFRVSLSSAVSSSSQGSGLDEGDALG-------

CqRCC1_22 --KDVC-------GGGCGFAMAI----------------SDTG-------

CqRCC1_23 --LVSA-------GASHS--VAL----------------LSGN-------

CqRCC1_24 --

CqRCC1_25 YSQMKG-------MGADTFRVSLSSAVSSSSQGSGLDEGDALG-------

CqRCC1_26 QMQIKA-------GRYHT--LL-----------------VSNS-------

CqRCC1_27 --

CqRCC1_28 --

CqRCC1_29 NFTGRN-------SSSESIRISSSSLVSSSSQGSFPDDFDSLG-------

CqRCC1_3 APAFVS-------RCRSFSSGV-------------TGNGSSLF-------

CqRCC1_30 NMQAKG-------SASDVFRVSVSSAPSTSSHGSAGDDCDALG-------

CqRCC1_31 --

CqRCC1_32 NGRSKC-------NGADTFRVSLSSAVSSSSHGSGHDDGDALG-------

CqRCC1_33 NGRSKC-------NGADTFRVSLSSAVSSSSHGSGHDDGDALG-------

CqRCC1_34 NMQLRGGTGEGFRLSADGFRLSISSTPTCSSQSSGPDDIESLG-------

CqRCC1_35 --

CqRCC1_36 -------------CCHV--AVL-----------------LVLC-------

CqRCC1_37 NFTGRN-------SSSESIRISSSSLVSSSSQGSFPDDFDSLG-------

CqRCC1_38 KSQAIA-------GPGHS--IAV----------------TSTG-------

CqRCC1_39 --

CqRCC1_4 --

CqRCC1_40 RRLLAA-------GADPDARDG-------------ESGWSSLHRALYFGH

CqRCC1_5 --

CqRCC1_6 --

CqRCC1_7 QMQIKA-------GRYHT--LL-----------------ISNS-------

CqRCC1_8 NMQLRGGTGEGFRLSADGFRLSISSTPSCSSQGSGPDDIESLG-------

CqRCC1_9 SGGGRL-------ESQRLFRAI-------------PSTARRAN-------

CqRCC1_1 -----KLI--TWGSTD----------DLGQS------------------

CqRCC1_10 -----DVY--IWGEVICE-----N--SFKAG------------------

CqRCC1_11 ---SLQLL--SWGRGS-S-----G--QLGSG------------------

CqRCC1_12 --

CqRCC1_13 FAVAGVLL--QSGASI-S-----I--EDFKS-----------------

CqRCC1_14 -----DLF--CWDTGF-S-----NSSIFGKIVYHNTLSPLTDLAVGDDQV

CqRCC1_15 -----V-EFME-------KVAKMTDAEELTV-----------------

CqRCC1_16 --

CqRCC1_17 ------RL--FWGA----------------------------------

CqRCC1_18 ------IY--VWGYNQ-R-----G--QTGRK-----------------

CqRCC1_19 ------AA--LWGNGD-F-----G--RLGLG-----------------

CqRCC1_2 -----TVY--SFGSNS-S-------GQLGHG-----------------

CqRCC1_20 -----VVC--SWGRGE-D-----G--QLGHG-----------------

CqRCC1_21 -----DVF--IWGEGTGD-----G--VLGGG-----------------

CqRCC1_22 -----KLI--TWGSTD----------DLGQS-----------------

CqRCC1_23 -----VVC--SWGRGE-D-----G--QLGHG-----------------

CqRCC1_24 ------AA--VWGNGD-F-----G--RLGLG-----------------

CqRCC1_25 -----DVF--IWGEGTGD-----G--VLGGG-----------------

CqRCC1_26 -----SVY--SCGSSL-C-------GVLGQG-----------------

CqRCC1_27 -----AVI--AWGSGE-DG-------QLGIG-----------------

CqRCC1_28 ------LL--FCGSTN-W--DTVG--RKKTG-----------------

CqRCC1_29 -----DVY--MWGEGISE-----G--IMGGG-----------------

CqRCC1_3 -----SVM--SFGDGS-Q-----G--ALGLP-----------------

CqRCC1_30 -----DVY--IWGEVICE-----N--SFKVG-----------------

CqRCC1_31 --

CqRCC1_32 -----DVY--IWGKA---------------------------------

CqRCC1_33 -----DVY--IWGEGTGD-----G--ILGGG-----------------

CqRCC1_34 -----DVY--VWGELWSD-----G--GLTDG-----------------

CqRCC1_35 -----L-VNGGRGSGE-DG-------QLGIG-----------------

CqRCC1_36 --

CqRCC1_37 -----DVF--MWGEGIGE-----G--IMGGG-----------------

CqRCC1_38 -----TVY--SFGSNS-S-------GQLGHG-----------------

CqRCC1_39 --

CqRCC1_4 ------LL--FCGSTN-W--DTVG--RKKTG-----------------

CqRCC1_40 FAVAGVLL--QSGASI-S-----I--EDFKL------------------

CqRCC1_5 --

CqRCC1_6 --

CqRCC1_7 -----SVY--SCGSSL-C-------GVLGQG------------------

CqRCC1_8 -----DMY--VWGELWSD-----G--GLTDG------------------

CqRCC1_9 -----DRY--LWN-----------------------------------

CqRCC1_1 ----------------YVTSG------KH-GE------TPEAFP------

CqRCC1_10 ----------------PDKSVNFVSTRAD-LL------LPKPLE------

CqRCC1_11 ----------------TE---------QT-RI------YPTPVA------

CqRCC1_12 ---LSP------

CqRCC1_13 ----------------RT---------PI-D-------------------

CqRCC1_14 CAVQARTGIIQCWEREGR---------LR-LL------SP-ALP------

CqRCC1_15 ----------------EE----------R-NL------LSVAYK------

CqRCC1_16 ---LPT------

CqRCC1_17 -------------------------------F------AP----------

CqRCC1_18 ----------------EK---------DQKLR------IPRQLPPELFGC

CqRCC1_19 ----------------SV---------DS-QW------RPVICP------

CqRCC1_2 ----------------LTPE----GNSAD-EY------QPRLIR------

CqRCC1_20 ----------------DA---------ED-RF------SPVQLS------

CqRCC1_21 ----------------VRRTGSCFGNKMD-SL------LPKALE------

CqRCC1_22 ----------------YVTSG------KH-GE------TPEAFP------

CqRCC1_23 ----------------DA---------ED-RF------SPVQLS------

CqRCC1_24 ----------------SV---------DS-QW------RPVICP------

CqRCC1_25 ----------------VRRTGSCFGNKMD-SL------LPKALE------

CqRCC1_26 ----------------SEMT----Q-----CG------SFSRIS------

CqRCC1_27 ----------------NN---------ED-KE------WVCQVK------

CqRCC1_28 ----------------IE---------G-NLY------SPTRLR------

CqRCC1_29 ----------------SLGV--SRSGKFD-AN------LPKALE------

CqRCC1_3 ----------------SS----LIGVGAD-AY------EPTVVP------

CqRCC1_30 ----------------PDKSVNFVSTRAD-LL------LPKPLE------

CqRCC1_31 --

CqRCC1_32 --

CqRCC1_33 ----------------AYRIESSLGVKLD-SC------RPKTLD------

CqRCC1_34 ----------------S---GNPNSTKVD-VL------TPKPLE------

CqRCC1_35 ----------------NN---------ED-KE------WVCQVK------

CqRCC1_36 -------------------------------LCPVLLTVPEIVH------

CqRCC1_37 ----------------SLGV--SPSGKYD-AN------LPKALE------

CqRCC1_38 ----------------LTPE----GNSAD-EY------RPRLIR------

CqRCC1_39 --

CqRCC1_4 ----------------IE---------G-NLY------SPTRLR------

CqRCC1_40 ----------------RT---------PI-D-------------------

CqRCC1_5 --

CqRCC1_6 --

CqRCC1_7 ----------------SEMT----Q-----CG------SFSRIS------

CqRCC1_8 ----------------S---GNPNSTKVD-VL------TPKPLE------

CqRCC1_9 --------------------------------------EPKLVK------

CqRCC1_1 -----------------------LPTEALIVRAAAG--WA-HCVSITD-G

CqRCC1_10 -S----------------------NVVLDVHLIACG--VR-HAALVTR-Q

CqRCC1_11 -SLIVPPSFRLPLSPGSLSPPLPEKSGVE-VGISCG--LF-HSAVL-I-D

CqRCC1_12 -R----------------------NPAHKIVAVAAG--EA-HTLALSG-D

CqRCC1_13 -----------------LL-SGPL-------SQILG--NQ-HASVT----

CqRCC1_14 -P----------------------VGETDFLSITSG--DGFSCGIVRD-E

CqRCC1_15 -----------------------NVI---------G--AR-R--------

CqRCC1_16 -R----------------------NPAHKIVAVAAG--EA-HTLALSG-D

CqRCC1_17 --------------------------NTVIRQVSCG--SV-HVVALSE-D

CqRCC1_18 PA----------------------GISTRWLDVACG--RE-HTAAVAS-D

CqRCC1_19 -A----------------------FNGDNLRSIACG--GA-HTLFLTE-S

CqRCC1_2 -----------------------SLQGIRIIQATAG--AG-RTMLISD-T

CqRCC1_20 -A----------------------LDGHDVNSITCG--AD-HTLAYSEPR

CqRCC1_21 -S----------------------AVVLDVQNIACG--GR-HAALVTK-Q

CqRCC1_22 -----------------------LPTEAPIVRAAAG--WA-HCVSITD-G

CqRCC1_23 -A----------------------LDGHDVNSITCG--AD-HTLAYSEPR

CqRCC1_24 -A----------------------FNGDNLRSIACG--GA-HTLFLTE-S

CqRCC1_25 -S----------------------AVVLDVQNIACG--VW-HAALVTK-Q

CqRCC1_26 -----------------------FPPSSPVVQVSAS--HN-HAAFVTL-S

CqRCC1_27 -----------------------ALQNYSVSSVVAG--SR-NSLALCE-D

CqRCC1_28 -P----------------------LVGIDIRFVASGCVSC-HCVALDV-D

CqRCC1_29 -S----------------------TVVLDVHHIACG--VR-HAVLVNR-Q

CqRCC1_3 -G----------------------L-PADVVSVSAG--HY-HSLAVTA-K

CqRCC1_30 -S----------------------NVVLDVYLIACG--VR-HAALVTR-Q

CqRCC1_31 -------------------------------ATETT--QQ-QAT-ELK-Q

CqRCC1_32 ----------------------------PEMNIACG--GR-HAALVTK-Q

CqRCC1_33 -S----------------------AVLLDVQNIACG--GR-HAALVTK-Q

CqRCC1_34 -S----------------------NVVLDVQQIACG--VS-HVALVTK-Q

CqRCC1_35 -----------------------ALQNYTVSSVVAG--SR-NSLALCE-D

CqRCC1_36 ---------------------------------------D-HVM----LR

CqRCC1_37 -S----------------------TVVLDVHHIACG--VR-HAVLVNR-Q

CqRCC1_38 -----------------------SLQGIRIIQATAG--AG-RTMLISD-T

CqRCC1_39 --

CqRCC1_4 -P----------------------LVGIDIRFVASGCVSC-HCVALDV-D

CqRCC1_40 -----------------LL-SGPL-------SQILG--NQ-HASVT----

CqRCC1_5 ---D-S

CqRCC1_6 -------------------------------ATETT--QK-HTK-ELK-Q

CqRCC1_7 -----------------------FPSSSPVVQVSAS--HN-HAAFVTQ-S

CqRCC1_8 -S----------------------NVVLDVQQIACG--VS-HVALVTK-Q

CqRCC1_9 -E----------------------LDNVKVQSAFAS--GV-ISAAIGD-D

CqRCC1_1 GEVY------TWG--WK-----------------------ECVPSGKVFG

CqRCC1_10 GEVF------TWG--E-----------------------ESGGRL-----

CqRCC1_11 GQFW------VWG--K-----------------------GDGGRL-----

CqRCC1_12 GKVY------SWG--R-----------------------GTFGRL-----

CqRCC1_13 TEVL------SWG--S-----------------------GTNYQL-----

CqRCC1_14 RNVL------CWG--E-----------------------NGIGDF-----

CqRCC1_15 ---ASWRIISSIEQKE-----------------------ESRGNE-----

CqRCC1_16 GKVY------SWG--R-----------------------GTFGRL-----

CqRCC1_17 GLLQ------SWG--Y-----------------------NEYSQL-----

CqRCC1_18 GSLF------TWG--SHLLPQIHPPAVLNLCGLWFMYRANDFGQL-----

CqRCC1_19 GRVY------ASG--L-----------------------NDFGQL-----

CqRCC1_2 GKVF------AFG--K-----------------------DSFGEA-----

CqRCC1_20 VQVY------SWG--W-----------------------GDFGRL-----

CqRCC1_21 GEIF------SWG--E-----------------------ETGGRL-----

CqRCC1_22 GEVY------TWG--WK-----------------------ECVPSGKVFG

CqRCC1_23 VQVY------SWG--W-----------------------GDFGRL-----

CqRCC1_24 GRVY------ASG--L-----------------------NDFGQL-----

CqRCC1_25 GEIF------SWG--E-----------------------ETGGRL-----

CqRCC1_26 GEVF------TCG--D-----------------------NSSFCC-----

CqRCC1_27 GKLF------TWG--W-----------------------NQRGTL-----

CqRCC1_28 GRCY------TWG--R-----------------------NERGQL-----

CqRCC1_29 GEIF------SWG--E-----------------------ESGGRL-----

CqRCC1_3 GELW------AWG--R-----------------------NDEGQL-----

CqRCC1_30 GEVF------TWG--E-----------------------ESGGRL-----

CqRCC1_31 QELW------SWG--A-----------------------GTEGQL-----

CqRCC1_32 GEVF------TWG--E-----------------------ESGGRL-----

CqRCC1_33 GEVF------TWG--E-----------------------ESGGRL-----

CqRCC1_34 GEVF------TWG--E-----------------------ESGGRL-----

CqRCC1_35 GKLF------TWG--W-----------------------NQRGTL-----

CqRCC1_36 LLVY------K---------------------------------------

CqRCC1_37 GEIF------SWG--E-----------------------ESGGRL-----

CqRCC1_38 GKVY------AFG--K-----------------------DSFGEA-----

CqRCC1_39 -KFW------VWG--K-----------------------GDGGRL-----

CqRCC1_4 GRCY------TWG--R-----------------------NEKGQL-----

CqRCC1_40 TEVL------SWG--S-----------------------GTNYQL-----

CqRCC1_5 GRLF------TCG--D-----------------------GSFGQL-----

CqRCC1_6 QELW------SWG--A-----------------------GTEGQL-----

CqRCC1_7 GEVF------TCG--D-----------------------NSSFCC-----

CqRCC1_8 GEVF------TWG--E-----------------------ESGGRL-----

CqRCC1_9 GSIW------TWG--R-----------------------SKRGQL-----

CqRCC1_1 DSSSGGSLEKDALEKQVSMFPEQVSPRGSKNASGSGSGTGFD--NKGAGE

CqRCC1_10 -------------------------------------GHGVG--K-----

CqRCC1_11 -------------------------------------GLGHE--D-----

CqRCC1_12 -------------------------------------GNGSQ--S-----

CqRCC1_13 -------------------------------------GTGNA--H-----

CqRCC1_14 --IE--R-----

CqRCC1_15 -------------------------------------DHVT--N------

CqRCC1_16 -------------------------------------GNGSQ--S-----

CqRCC1_17 -------------------------------------GRGFT--C-----

CqRCC1_18 -------------------------------------GDGTE--E-----

CqRCC1_19 -------------------------------------GISDD--K-----

CqRCC1_2 -------------------------------------EYGVQGSR-----

CqRCC1_20 -------------------------------------GHGNS--T-----

CqRCC1_21 -------------------------------------GHGVD--A-----

CqRCC1_22 DSSSGGGLEKEALEKSSSMFPEQVSPRGSKNASGSGLGTGFD--NKGAGE

CqRCC1_23 -------------------------------------GHGNS--T-----

CqRCC1_24 -------------------------------------GISDD--K-----

CqRCC1_25 -------------------------------------GHGVD--A-----

CqRCC1_26 -------------------------------------GHKDT-GR-----

CqRCC1_27 -------------------------------------GQPPE-T------

CqRCC1_28 -------------------------------------GHGDT--L-----

CqRCC1_29 -------------------------------------GHGVE--V-----

CqRCC1_3 -------------------------------------GRGLL--E-----

CqRCC1_30 -------------------------------------GHGVG--K-----

CqRCC1_31 -------------------------------------GTGNL--K-----

CqRCC1_32 -------------------------------------GHGVD--S-----

CqRCC1_33 -------------------------------------GHGVD--S-----

CqRCC1_34 -------------------------------------GHGIE--R-----

CqRCC1_35 -------------------------------------GQPPE-T------

CqRCC1_36 --

CqRCC1_37 -------------------------------------GHGVE--V-----

CqRCC1_38 -------------------------------------EYGAQGSR-----

CqRCC1_39 -------------------------------------GLGHE--D-----

CqRCC1_4 -------------------------------------GHGDT--L-----

CqRCC1_40 -------------------------------------GTGNA--H-----

CqRCC1_5 -------------------------------------GHGDY--Q-----

CqRCC1_6 -------------------------------------GTGNL--K-----

CqRCC1_7 -------------------------------------GHKDT-GR-----

CqRCC1_8 -------------------------------------GHGIE--R-----

CqRCC1_9 -------------------------------------GHGKG--I-----

CqRCC1_1 ETAKRRRVSSAKQAAENSTTSEETLS-A-LPCL-----------------

CqRCC1_10 D------------------------V-F-QPRL-----------------

CqRCC1_11 S------------------------V-F-VPTLN--------PHLS----

CqRCC1_12 D------------------------E-K-FPVPI--------KWDNDGDL

CqRCC1_13 I------------------------Q-K-LPCKL--------DALHGS--

CqRCC1_14 ---EFGSL--

CqRCC1_15 I-----------------------KE-Y-RGKI------------------

CqRCC1_16 D------------------------E-N-HPVPI--------KWDNND--

CqRCC1_17 E------------------------GLQ-GPGVVKAYAKHLDEAPERV--

CqRCC1_18 S------------------------R-K-YPKKV--------DLLDTE--

CqRCC1_19 N-----------------------Y-AMEPAE----------------

CqRCC1_2 I------------------------VN-TPQL----------------

CqRCC1_20 D-----------------------L-F-IPHPI--------KTLDGV--

CqRCC1_21 D-----------------------V-S-HPKL----------------

CqRCC1_22 ETAKRRRVSSAKQAAESSTTSEETLS-A-LPCL----------------

CqRCC1_23 D-----------------------L-F-IPHPI--------KTLDGV--

CqRCC1_24 N-----------------------Y-AMEPVE----------------

CqRCC1_25 D-----------------------V-S-HPKL----------------

CqRCC1_26 P------------------------IF-RPRL----------------

CqRCC1_27 K----------------------AE-N-VPTQ----------------

CqRCC1_28 Q-----------------------R-D-RPTVI--------SELSKH--

CqRCC1_29 D-----------------------V-S-NPKL----------------

CqRCC1_3 PRC---------------------SW-N-EPKLV--------KELDNV--

CqRCC1_30 D-----------------------V-F-QPRL----------------

CqRCC1_31 D-----------------------E-L-TPQL----------------

CqRCC1_32 D-----------------------V-S-HPKL----------------

CqRCC1_33 D-----------------------V-S-HPKL----------------

CqRCC1_34 D-----------------------F-T-RPHL----------------

CqRCC1_35 K----------------------AE-N-VPTQ----------------

CqRCC1_36 --

CqRCC1_37 D-----------------------V-S-NPKL----------------

CqRCC1_38 I------------------------VN-TPQL----------------

CqRCC1_39 S-----------------------V-F-VPTLN--------PHLT----

CqRCC1_4 Q-----------------------R-D-RPTVI--------SELSKH--

CqRCC1_40 I-----------------------Q-K-LPCKL--------DALHGS--

CqRCC1_5 S-----------------------C-S-SPME----------------

CqRCC1_6 D-----------------------E-F-TPQL----------------

CqRCC1_7 P------------------------IF-RPRL----------------

CqRCC1_8 D-----------------------F-T-RPHL----------------

CqRCC1_9 --

CqRCC1_1 --

CqRCC1_10 --

CqRCC1_11 ------GVKGLALGGLHSVALT-------------------SLGQIFTWG

CqRCC1_12 GNEKCPKFVGVAAGAYHSLALAGENVSVPRQLDMLV--------------

CqRCC1_13 ------FIKLISASKFHSVAVT-------------------GRGELYTWG

CqRCC1_14 --

CqRCC1_15 --

CqRCC1_16 ------DLQDEKC-------------------------------------

CqRCC1_17 ------KITQ--

CqRCC1_18 ------YVKSVSCGANCTAAIAEPR----------KNDGTISTGRLWVWG

CqRCC1_19 --

CqRCC1_2 --

CqRCC1_20 --

CqRCC1_21 --

CqRCC1_22 --

CqRCC1_23 --

CqRCC1_24 --

CqRCC1_25 --

CqRCC1_26 --

CqRCC1_27 --

CqRCC1_28 --

CqRCC1_29 --

CqRCC1_3 ------KVQSAFASGVISAAIG-------------------DDGSIWTWG

CqRCC1_30 --

CqRCC1_31 --

CqRCC1_32 --

CqRCC1_33 --

CqRCC1_34 --

CqRCC1_35 --

CqRCC1_36 --

CqRCC1_37 --

CqRCC1_38 --

CqRCC1_39 ------GVKGLALGGLHSVALT-------------------SLGQIFTWG

CqRCC1_4 --

CqRCC1_40 ------FIKLISASKFHSVAVS-------------------GRGELYTWG

CqRCC1_5 --

CqRCC1_6 --

CqRCC1_7 --

CqRCC1_8 --

CqRCC1_9 --

CqRCC1_1 ------------------------------VTLSPG-----VRISTVAAG

CqRCC1_10 ------------------------------VESLVA-----YNVDFVACG

CqRCC1_11 YGGFGA---LGHSVF------TRELIPKL-VEGPWT-----EKIKHISTS

CqRCC1_12 -----------------QLNSPRSLGDDSEGKSKNP-----LKVCAIEAG

CqRCC1_13 FGRGGR---LGHPEFDIHSGQAAVITPRQVISGLGS-----RRVKAVAAA

CqRCC1_14 --SMLSVVAG

CqRCC1_15 ------------------------------ETELSK-----ICDGILNLL

CqRCC1_16 ---PKFVGVAAG

CqRCC1_17 --VSCG

CqRCC1_18 QNQGSNYPRLFWGSFAPN-----------------------TVIRQVSCG

CqRCC1_19 ------------------------------VFGI-E-----KEIKHVSAG

CqRCC1_2 ------------------------------VESLKD-----IFVVQAAIG

CqRCC1_20 --KIKQIACG

CqRCC1_21 ------------------------------IDALST-----TNIELVACG

CqRCC1_22 ------------------------------VTLSPG-----VRISTVAAG

CqRCC1_23 --KIKQIACG

CqRCC1_24 ------------------------------VVGI-E-----KEIKHVSAG

CqRCC1_25 ------------------------------IDALST-----TNIELVACG

CqRCC1_26 ------------------------------VEALKG-----ISCKQVAAG

CqRCC1_27 ------------------------------VKALAN-----VKIVQAAIG

CqRCC1_28 --RIVKADAG

CqRCC1_29 ------------------------------IEGLSG-----MNIELVACG

CqRCC1_3 RSKRGQ---LGHGKGI-----TESLVPSK-VKALTG-----EHITKVSLG

CqRCC1_30 ------------------------------VESLVA-----YNVDFVACG

CqRCC1_31 ------------------------------LINLSNPLSSFGQISLLSCG

CqRCC1_32 ------------------------------VDALKS-----TNVELVACG

CqRCC1_33 ------------------------------VDALKS-----TNIELVACG

CqRCC1_34 ------------------------------VEFLAV-----STVDFVACG

CqRCC1_35 ------------------------------VKALAN-----VKIVQAAIG

CqRCC1_36 --

CqRCC1_37 ------------------------------IEGLSG-----MNIELVACG

CqRCC1_38 ------------------------------VESLKD-----IFVVQAAIG

CqRCC1_39 YGGFGA---LGHSVF------TRELIPRL-VEGPWT-----EKIKHISTS

CqRCC1_4 --RIVKADAG

CqRCC1_40 FGRGGR---LGHPEFDIHSGQAAVITPRQVVSGLGS-----RRVKAVAAA

CqRCC1_5 --

CqRCC1_6 --

CqRCC1_7 ------------------------------VEALKG-----ISCKQVAAG

CqRCC1_8 ------------------------------VEFLAV-----TTVDFVACG

CqRCC1_9 ---------------------TESLVPFK-VEALTG-----EHITKVSLG

CqRCC1_1 GRHTLALSASDV------------------GQVWGWGYGG--EGQLGLGS

CqRCC1_10 EFHSCAVTMS--------------------GELYTWGDGTHNAGLLGHGN

CqRCC1_11 GTHTAAVSES--------------------GEIYTWGREE-GDGRLGRGP

CqRCC1_12 GMMSLAIDNL--------------------GALWMWGNIP--QQRSPDDD

CqRCC1_13 KHHTVIATES--------------------GEVFTWGSNR--EGQLGYAS

CqRCC1_14 KSHVCGVAYS--------------------GLLICKGLND--SGQLDVPS

CqRCC1_15 ESHLIPSASS--------------------AESKVFYQ------------

CqRCC1_16 AYHSLALAGT-LAQLNSPRSLGDDSEGKSKNPLKMWGNIP--QQSSPDDN

CqRCC1_17 EYHTAAVSET--------------------GDVYTWGLG-----------

CqRCC1_18 SVHVVALSED--------------------GLLQSWGYNE--YSQLGRGF

CqRCC1_19 YCHSCAITGA--

CqRCC1_2 NFFTAVLSRE--------------------GRVYTFSWGS--DGKLGHQT

CqRCC1_20 DSHCLAVTMD--------------------GEVASWGRNQ--NGQLGIGT

CqRCC1_21 EHHTCAVTLS--------------------GELYTWGDGAFNFGLLGHGN

CqRCC1_22 GRHTLALSASDV------------------GQVWGWGYGG--EGQLGLGS

CqRCC1_23 DSHCLAVTMD--------------------GVVASWGRNQ--NGQLGIGT

CqRCC1_24 YCHSCAITVD--------------------GELYMWGKNS--NGQLGLGK

CqRCC1_25 EHHTCAVTLS--------------------GELYTWGDGAFNFGLLGHGN

CqRCC1_26 LSFTMFLTRE--------------------GHVYTCGTNT--HGQLGHGD

CqRCC1_27 GWHCLAVDDQ--------------------GRAYAWGGNE--YGQCGEDP

CqRCC1_28 RSHTVVMADD--------------------GQSFAFGWNK--HGQLGTGS

CqRCC1_29 EYHTCAITTS--------------------GDLFTWGGGTWNPGMLGHVS

CqRCC1_3 WGHVLAQTID--------------------GKLFGWGYSA--DGRLGRLG

CqRCC1_30 EFHSCAVTMS--------------------GELYTWGDGTHNAGLLGHGN

CqRCC1_31 GAHVIAITSG--------------------GKVLSWGRGA--SGQLGLGQ

CqRCC1_32 ENHSCAVSLS--------------------GDLYIWGGRKYSFGLSGQGD

CqRCC1_33 ENHSCAVSLS--------------------GDLYIWGGRKYSFGLSGQGD

CqRCC1_34 EYHSCAVSTS--------------------GDVFTWGDGIHHAGLLGHGT

CqRCC1_35 GWHCLAVDDQ--------------------GRAYAWGGNE--YGQCGEDP

CqRCC1_36 --

CqRCC1_37 EYHTCAITTS--------------------GDLFTWGGGTWNPGMLGHVS

CqRCC1_38 NFFTAVLSRE--------------------GRVYTFSWGS--DGKLGHQT

CqRCC1_39 GTHTAAVSVS--------------------GEIYTWGREE-GDGRLGRGP

CqRCC1_4 RSHTVVMTDD--------------------GQSFAFGWNK--HGQLGSGS

CqRCC1_40 KHHTVIATES--------------------GEVFTWGSNR--EGQLGYAS

CqRCC1_5 --

CqRCC1_6 --

CqRCC1_7 LSFTMFLTRE--------------------GHVYTCGTNT--HGQLGHGD

CqRCC1_8 EYHSCAVSTS--------------------GDVFTWGDGIHHAGLLGQGT

CqRCC1_9 WGHVLAQTVD--------------------GKLFGWGYSA--DGRLGRLG

CqRCC1_1 R-I--RMV

CqRCC1_10 E-V--S--H-

CqRCC1_11 G-R---------------------GPDQTG---------------G--L-

CqRCC1_12 TFT--LI-

CqRCC1_13 V-D--T--Q-

CqRCC1_14 E-K---

CqRCC1_15 --

CqRCC1_16 TFT--LI-

CqRCC1_17 --

CqRCC1_18 TCE--GL-

CqRCC1_19 ---PSAV-

CqRCC1_2 D-P--ND-

CqRCC1_20 A-E--DS-

CqRCC1_21 D-V--S--H-

CqRCC1_22 R-I--RMV

CqRCC1_23 A-E--DS-

CqRCC1_24 G-A--LSAV-

CqRCC1_25 D-V--S--H-

CqRCC1_26 T-M--DR-

CqRCC1_27 E-K--DET

CqRCC1_28 -LR--NE-

CqRCC1_29 E-A--G--H-

CqRCC1_3 E-TVKASPLDSSAELPGRHEMTSSEALDVAEKLVLESMEKEKDMPI--V-

CqRCC1_30 E-V--S--H-

CqRCC1_31 I-M--P-NS-

CqRCC1_32 E-M--S--H-

CqRCC1_33 E-M--S--H-

CqRCC1_34 D-A--C--H-

CqRCC1_35 E-K--DET

CqRCC1_36 --

CqRCC1_37 E-A--G--H-

CqRCC1_38 D-P--ND-

CqRCC1_39 G-R---------------------GPDQTG---------------G--L-

CqRCC1_4 -LR--NV-

CqRCC1_40 V-D--T--Q-

CqRCC1_5 --

CqRCC1_6 --

CqRCC1_7 T-M--DR-

CqRCC1_8 D-A--C--H-

CqRCC1_9 E-TVKASPLDSSAELPGRHEMTSSEALDVAEKHVLESMEKEKDMPI--V-

CqRCC1_1 --S------S-----------------------------PHPV-PCIESS

CqRCC1_10 --W------I-----------------------------PKRI-A-----

CqRCC1_11 --S------V-----------------------------PGKV-------

CqRCC1_12 --S------V-----------------------------PDPVPM-----

CqRCC1_13 --P------T-----------------------------PRRV-------

CqRCC1_14 --

CqRCC1_15 --

CqRCC1_16 --S------V-----------------------------PNPIPM-----

CqRCC1_17 --

CqRCC1_18 --Q------GPGIVKAY----------------------AKHLD------

CqRCC1_19 --L------R-----------------------------PLKV-------

CqRCC1_2 --V------Q-----------------------------PRPL-L-----

CqRCC1_20 --L------I-----------------------------PQKI-------

CqRCC1_21 --W------V-----------------------------PKRV-N-----

CqRCC1_22 --S------S-----------------------------PHPV-PCIESS

CqRCC1_23 --L------I-----------------------------PQKI-------

CqRCC1_24 --S------R-----------------------------PLKI-------

CqRCC1_25 --W------V-----------------------------PKRV-N-----

CqRCC1_26 --P------M-----------------------------PKLV-------

CqRCC1_27 GRLLRRDIVI-----------------------------PQRC-------

CqRCC1_28 --I------E-----------------------------SSPIR------

CqRCC1_29 --W------I-----------------------------PKRV-S-----

CqRCC1_3 --W------E-----------------------------PHLI-------

CqRCC1_30 --W------I-----------------------------PKRI-A-----

CqRCC1_31 --L------Y-----------------------------PKLV-------

CqRCC1_32 --W------I-----------------------------PKRL-I-----

CqRCC1_33 --W------I-----------------------------PKRL-I-----

CqRCC1_34 --W------I-----------------------------PKRI-S-----

CqRCC1_35 GRLLRRDIVI-----------------------------PQRC-------

CqRCC1_36 --

CqRCC1_37 --W------I-----------------------------PKRV-S-----

CqRCC1_38 --V------Q-----------------------------PRPL-L-----

CqRCC1_39 --S------V-----------------------------PGKV-------

CqRCC1_4 --M------QCRGMGELCGYLMASDMVVDLWDLSLKEIESSPIR------

CqRCC1_40 --P------T-----------------------------PRRV-------

CqRCC1_5 --V-------

CqRCC1_6 --L-------

CqRCC1_7 --P------M-----------------------------PKLV-------

CqRCC1_8 --W------I-----------------------------PKRI-S-----

CqRCC1_9 --W------E-----------------------------PHLL-------

CqRCC1_1 YRKDRHSGF-QGGLNSDGHGFRVPGS-YIKGI-----------------

CqRCC1_10 --------------------GPLEGL-QVASV-----------------

CqRCC1_11 --------------------KGMP-V-PAAAV-----------------

CqRCC1_12 --------------------FDFYGH-TVVKV-----------------

CqRCC1_13 --------------------SSLK-S-KIVAV-----------------

CqRCC1_14 ------------------------PF-HFLEL-----------------

CqRCC1_15 -------------------------------KMKGDYHRYLAEFKTGAER

CqRCC1_16 --------------------FDFYGH-TVVKV------------------

CqRCC1_17 --

CqRCC1_18 --------------------EAPERV-KIIQV------------------

CqRCC1_19 --------------------GYLTGI-IIEMA------------------

CqRCC1_2 --------------------GALENI-PVVQI------------------

CqRCC1_20 --------------------QAFQGI-PVKMV------------------

CqRCC1_21 --------------------GPLEGI-HVSYI------------------

CqRCC1_22 YRKDGHSGFSQGGLNSDGHGFRVPGS-YIKGI------------------

CqRCC1_23 --------------------QAFQGI-PVKMV------------------

CqRCC1_24 --------------------GYLTGI-IIEMA------------------

CqRCC1_25 --------------------GPLEGI-HVSYI------------------

CqRCC1_26 --------------------ELLESVGSIVQI------------------

CqRCC1_27 ----------------------APKL-KVRQV------------------

CqRCC1_28 ----------------------CQVS-EVKAV------------------

CqRCC1_29 --------------------GPLEGM-HVSFA------------------

CqRCC1_3 --------------------EELHSI-SVTDI------------------

CqRCC1_30 --------------------GPLEGL-QVASV------------------

CqRCC1_31 --------------------DSLTNF-NISHV------------------

CqRCC1_32 --------------------GPLEGI-HVSSI------------------

CqRCC1_33 --------------------GPLEGI-HVSSI------------------

CqRCC1_34 --------------------GPLEGL-QVISI------------------

CqRCC1_35 ----------------------APKL-KVRQV------------------

CqRCC1_36 ----------------------------FVML------------------

CqRCC1_37 --------------------GPLEGM-YVSFA------------------

CqRCC1_38 --------------------GALENI-PVVQI------------------

CqRCC1_39 --------------------KGMP-V-PAAAV------------------

CqRCC1_4 ----------------------CQVS-EVKAV------------------

CqRCC1_40 --------------------SSLK-S-KIVAV------------------

CqRCC1_5 --------------------SFFNSR-HVEQI------------------

CqRCC1_6 --------------------INLSNS------------------------

CqRCC1_7 --------------------ELLESVGSIVQI------------------

CqRCC1_8 --------------------GPLEGL-QVISI------------------

CqRCC1_9 --------------------EELHSI-PVTDI------------------

CqRCC1_1 -------------ACGGR--------------------------------

CqRCC1_10 -------------TCGPW--------------------------------

CqRCC1_11 -------------ACGGF--------------------------------

CqRCC1_12 -------------ACGNE--------------------------------

CqRCC1_13 -------------AAANK--------------------------------

CqRCC1_14 -------------ALSGT--------------------------------

CqRCC1_15 KEAAENTLILLLLLKLPPTHPIR---LGLALNFSVFYYEILNSPDR-----

CqRCC1_16 -------------ACANE--------------------------------

CqRCC1_17 --

CqRCC1_18 -------------SCGEY--------------------------------

CqRCC1_19 -------------ALGSE--------------------------------

CqRCC1_2 -------------AAGYC--------------------------------

CqRCC1_20 -------------AAGAE--------------------------------

CqRCC1_21 -------------SCGPW--------------------------------

CqRCC1_22 -------------ACGGR--------------------------------

CqRCC1_23 -------------AAGAE--------------------------------

CqRCC1_24 -------------ALGSE--------------------------------

CqRCC1_25 -------------SCGPW--------------------------------

CqRCC1_26 -------------SAGPS--------------------------------

CqRCC1_27 -------------AAGGTHSVVLTREGHVWTWGQ----PWPPGDIKQIFV

CqRCC1_28 -------------ACGGE--------------------------------

CqRCC1_29 -------------SCGPW--------------------------------

CqRCC1_3 -------------SCGLD--------------------------------

CqRCC1_30 -------------TCGPW--------------------------------

CqRCC1_31 -------------SAGWN--------------------------------

CqRCC1_32 -------------SCGPW--------------------------------

CqRCC1_33 -------------SCGPW--------------------------------

CqRCC1_34 -------------ACGTW--------------------------------

CqRCC1_35 -------------AAGGTHSVVLTREGHVWTWGQ----PWPPGDIKQIFV

CqRCC1_36 -------------DYKTL--------------------------------

CqRCC1_37 -------------SCGPW--------------------------------

CqRCC1_38 -------------AAGYC--------------------------------

CqRCC1_39 -------------ACGGF--------------------------------

CqRCC1_4 -------------ACGGE--------------------------------

CqRCC1_40 -------------AAANK--------------------------------

CqRCC1_5 -------------ACGMR--------------------------------

CqRCC1_6 --

CqRCC1_7 -------------SAGPS--------------------------------

CqRCC1_8 -------------ACGTW--------------------------------

CqRCC1_9 -------------SCGLD--------------------------------

CqRCC1_1 ------------------HS-AAIT---D--AGA----------ILAFGW

CqRCC1_10 ------------------HT-ALIT-S----TG--Q--------LFTFGD

CqRCC1_11 ------------------FT-MALT-E----EG--K--------LWCWGA

CqRCC1_12 ------------------HV-VALV-S----AG--EKHIGDDLVCYTWGN

CqRCC1_13 ------------------HT-AAVS-E----TG--E--------VFTWGC

CqRCC1_14 ------------------YSCALQR-N----NG--S--------VICWGN

CqRCC1_15 -------------ACNLAKQ------------------------AFDEAI

CqRCC1_16 ------------------HV-VALV-S----AG--EKHVGDDLVCYTWGN

CqRCC1_17 --

CqRCC1_18 ------------------HT-AAVS-E----TG--D--------VYTWGL

CqRCC1_19 ------------------HS-IAVT-D----EG--S--------TLSWGM

CqRCC1_2 ------------------YL-LALACQ----PGGMS--------VYSVGC

CqRCC1_20 ------------------HS-AAVT-E----SG--E--------VYGWGW

CqRCC1_21 ------------------HT-AVVT-S----AG--Q--------LFTFGD

CqRCC1_22 ------------------HS-AAIT---D--AGA----------ILAFGW

CqRCC1_23 ------------------HS-AAVT-E----SG--E--------VYGWGW

CqRCC1_24 ------------------HS-IAVT-D----EG--A--------TLSWGM

CqRCC1_25 ------------------HT-AVVT-S----AG--Q--------LFTFGD

CqRCC1_26 ------------------YS-IAVA-H----DG--T--------VYSFGS

CqRCC1_27 PVRVQGPEKVRLIAVGAFHN-LALE---ED--G--T--------LWAWGN

CqRCC1_28 ------------------FT-AWLS-STE--GA--T--------ILTAGL

CqRCC1_29 ------------------HT-AVVL-S----TG--Q--------LFTFGD

CqRCC1_3 ------------------HS-LLIC-R----DN--T--------ILSGGS

CqRCC1_30 ------------------HT-ALIT-S----TG--Q--------LFTFGD

CqRCC1_31 ------------------HS-GFVS-D----SG--R--------LFTCGD

CqRCC1_32 ------------------HT-AIVT-S----AG--Q--------LFTFGD

CqRCC1_33 ------------------HT-AIVT-S----AG--Q--------LFTFGD

CqRCC1_34 ------------------HS-ALTA-S----NG--K--------LFTFGD

CqRCC1_35 PVRVQGPEKVRLIAVGAFHN-LALE---ED--G--T--------LWAWGN

CqRCC1_36 ------------------QP-YRLR-K----NS--A--------LSLFGE

CqRCC1_37 ------------------HT-AVVL-S----TG--Q--------LFTFGD

CqRCC1_38 ------------------YL-LALACL----PGGMS--------VYSVGC

CqRCC1_39 ------------------FT-MALT-E----EG--K--------LWCWGA

CqRCC1_4 ------------------FT-AWLS-STE--GA--T--------ILTAGL

CqRCC1_40 ------------------HT-AAVS-E----IG--E--------VFTWGC

CqRCC1_5 ------------------HS-LVLL-K-GNLGD--K--------VFGFGS

CqRCC1_6 --LSSFG-

CqRCC1_7 ------------------YS-IAVA-H----DG--T--------VYSFGS

CqRCC1_8 ------------------HS-ALTT-S----NG--K--------LFTFGD

CqRCC1_9 ------------------HS-LLIC-R----DN--T--------MLSGGS

CqRCC1_1 GL-YGQCGQGAT---------DD--------------VLSPTCVSSL--L

CqRCC1_10 GT-FGVLGHGDR---------EN--------------IPYPREVDSL--S

CqRCC1_11 NF-NYELGTGDK---------VG--------------SWKPRLNSSL--E

CqRCC1_12 NN-HGQLGLGDT---------ES--------------RSHPQAVAQF--S

CqRCC1_13 NK-EGQLGYGTS---------NS--------SS----NNTPRLVEYL--K

CqRCC1_14 GG-NT-----------------S--------------AITDQKIQK----

CqRCC1_15 SE-LDTLGEDSY---------KD-----------------STLIMQL--L

CqRCC1_16 NN-HGQLGLGDT---------ES--------------RSHPQAVAQF--S

CqRCC1_17 -N-MGQLGHCYL---------QSE-----------DKELIPRRVVAL--D

CqRCC1_18 GN-MGQLGHCSL---------QSE-----------DKELIPRRVVAL--D

CqRCC1_19 GS-SGRLGHDRQSGLFNFLSSRS--------------NYTPRLIKRL--E

CqRCC1_2 GL-GGKLGHGSR---------TD--------------EKYPRLIEQFQTL

CqRCC1_20 GR-YGNLGLGDR---------KD--------------RLVPERSSSI--D

CqRCC1_21 GT-FGVLGHGDR---------KS--------------VSIPREVESL--K

CqRCC1_22 GL-YGQCGQGAT---------DD--------------VLSPTCVSSL--L

CqRCC1_23 GR-YGNLGLGDR---------KD--------------RLVPERSSSI--D

CqRCC1_24 GS-SGRLGHDRQSGLFNFLSSRS--------------EYTPRLIKRL--E

CqRCC1_25 GT-FGVLGHGDR---------KS--------------VSIPREVESL--K

CqRCC1_26 GT-NFCLGHGEQ---------RN--------------EHQPRAILSFRRK

CqRCC1_27 NE-YGQLGTGDT---------QP--------------RSQPIRVQGL--S

CqRCC1_28 PQ-YGQLGHGTD---------NEYNTKDSSVRLAYEAQPRPKAIASF--S

CqRCC1_29 GT-FGVLGHGDR---------MS--------------YTVPREVEAL--K

CqRCC1_3 NI-YGQLGRENQ---------GL--------------RFFPVDIS-----

CqRCC1_30 GT-FGVLGHGDR---------EN--------------IPYPREVDSL--S

CqRCC1_31 GS-FGQLGHGDY---------QS--------------CSSPMEVSFF--N

CqRCC1_32 GT-FGVLGHGDR---------KS--------------FSAPREVESL--K

CqRCC1_33 GT-FGVLGHGDR---------KS--------------FSAPREVESL--K

CqRCC1_34 GS-FGVLGHGDR---------ES--------------VSYPKEVQLL--S

CqRCC1_35 NE-YGQLGTGDT---------QP--------------RSQPIRVQGL--S

CqRCC1_36 GTKIGQLGIGTA---------ED--------------SLIPQKIQAF--Q

CqRCC1_37 GT-FGVLGHGDR---------MS--------------YTVPREVEAL--K

CqRCC1_38 GL-GGKLGHGSR---------TD--------------EKYPRLIEQFQTL

CqRCC1_39 NS-NYELGTGDK---------VG--------------GWQPRLNSYL--E

CqRCC1_4 PQ-YGQLGHGTD---------NEYNTKDSSVRLAYEPQPRPKAIASF--S

CqRCC1_40 NK-EGQLGYGTS---------NS--------SS----NNTPRLVEYL--K

CqRCC1_5 GK-RGQLGVYEDK-------VKS--------------VSIPKTCS-----

CqRCC1_6 --

CqRCC1_7 GT-NFCLGHGEQ---------RN--------------EHQPRAILSFRRK

CqRCC1_8 GS-FGVLGHGER---------ES--------------VSYPKEVQLL--S

CqRCC1_9 NI-YGQLGRENQ---------GL--------------GFFPVDIS-----

CqRCC1_1 ---GIRIETAAA----------GLWHTLCIS--------------TEG-D

CqRCC1_10 ---GLRTIAVAC----------GVWHTAAVVEVIVTQS---SSSISSG-K

CqRCC1_11 ---DMRVVQIAC----------GGYHSLALTE--------------GG-K

CqRCC1_12 EGSAWRAYDVAC----------GSFHTVVLTLKKQ------PSDTLAS-V

CqRCC1_13 ---GKFFMAVSA----------AKYHTVVLGA--------------DG-E

CqRCC1_14 ----LSFESIIS----------GLDYTCGLR--------------SDNFT

CqRCC1_15 ---RDN-----LTLWTSDNAEEGGDEI--KE--------------TAG-K

CqRCC1_16 EGSAWRAYDVAC----------GSFHTVVLTFKKQ------PSDTLAS-V

CqRCC1_17 ---GIHITSVAC----------GGVHTCAVT--------------AKG-A

CqRCC1_18 ---GIYITSVAC----------GGVHTCAVT--------------AKG-A

CqRCC1_19 ---GIKVKTVAA----------GLLHSACIDE--------------NG-N

CqRCC1_2 ---NIQPMVVAA----------GAWHAAVVG--------------KDG-R

CqRCC1_20 ---GIKMIQVAC----------GWRHTITVT--------------SSG-S

CqRCC1_21 ---GLRTVRAAC----------GVWHTAAVVEVMVGSSG--SSNCSSG-K

CqRCC1_22 ---GIRIEAAAA----------GLWHTLCKS--------------TEG-D

CqRCC1_23 ---GIKMIQVAC----------GWRHTITVT--------------SSG-S

CqRCC1_24 ---GIKVKTVAA----------GLLHSACIDE--------------NG-N

CqRCC1_25 ---GLRTVRAAC----------GVWHTAAVVEVMVGSSG--SSNCSSG-K

CqRCC1_26 ---GIHVVRVSA----------GDEHVVALD--------------SCG-Y

CqRCC1_27 ---GLTLVDIAA----------GGWHSTALT--------------SAG-E

CqRCC1_28 ---GETIVKVAC----------GTNHSVAVD--------------SNG-Y

CqRCC1_29 ---GQRTLRVAC----------GAWHTAAVVDILESETMFDRSFLASG-K

CqRCC1_3 ----FRPLSVAC----------GLGHSLAICEIPASED---GN-DFRK-A

CqRCC1_30 ---GLRTIAVAC----------GVWHTAAVVEVIVTQS---SSSISSG-K

CqRCC1_31 ---SKHVEQIAC----------GMRHSLVLLKGN-----------LGD-K
CqRCC1_32 ---GLRTVRAAC----------GVWHTAAVVEVMVGNSS--SSNCSSG-K
CqRCC1_33 ---GLRTVRAAC----------GVWHTAAVVEVMVGNSS--SSNCSSG-K
CqRCC1_34 ---GLKTVKVAC----------GVWHTAAIVEVMSPSTS--GSSISSR-K
CqRCC1_35 ---GLTLVDIAA----------GGWHSTALT--------------SAG-E
CqRCC1_36 ---GIPVKMVAA----------GAEH------------------------
CqRCC1_37 ---GQRTLRVAC----------GAWHTAAVVDILESETMFDRSFLASG-K
CqRCC1_38 ---NIQPMVVAA----------GAWHAAVVG--------------KDG-R
CqRCC1_39 ---DMRIVQIAC----------GGYHSLALTE--------------GG-K
CqRCC1_4 ---GETIVKVAC----------GTNHSVAVD--------------SNG-Y
CqRCC1_40 ---GKFFTAVSA----------AKYHTVVLGA--------------DG-E
CqRCC1_5 --
CqRCC1_6 --
CqRCC1_7 ---GIHVVRVSA----------GDEHVVALD--------------SCG-Y
CqRCC1_8 ---GLKTVKVAC----------GVWHTAAIVEVMSPSTS--GSSISSR-K
CqRCC1_9 ----FRPLSVAC----------GLGHSLAICEMPASED---DN-DFRK-A

CqRCC1_1 VYAFGGNQFGQLGTGSDQ-A-----------ETL----------------
CqRCC1_10 LFTWGDGDKNRLGQGDKE-P---------R--LKPTCVPS---LI-----
CqRCC1_11 VLSWGHGGHG--
CqRCC1_12 CWTFGLGENGQLGHGTTQ-S---------T--SLPEPARA---LP-----
CqRCC1_13 VFTWGHRLVT------------------------PRRVIIARNLKKNGNA
CqRCC1_14 VNCWGPGWSGKLDTSLSL-P---------S--MIPG-------KC-----
CqRCC1_15 SGEQGSGEDGQLGIGNNED------------KEWVCQVKA---LQ-----
CqRCC1_16 CWTFGLGENGQLGHGTTQ-S---------S--SLPEPARE---LP-----
CqRCC1_17 LYAWGGGQVGQLGLGPQT-SSASFVFKDSL--LMLRNILA---LV-----
CqRCC1_18 LYAWGGGQVGQLGLGPQT-SSASFVFKDSL--LMLRNIPA---LV-----
CqRCC1_19 VFIFGERRINKLNFGNSD-N---------I--TGPSMMTE---LP-----
CqRCC1_2 VCTWGWGRYGCLGHGNED-C-----------ESAPKVVEG---LT-----
CqRCC1_20 IYTYGWSKYGQLGHGDFE-D---------H--LTPYKLEA---LS-----

CqRCC1_21 LFTWGDGDKGRLGHGDKE-A---------K--LVPTCVAD---LV-----

CqRCC1_22 VYAFGGNQFGQLGTGSDQ-A-----------ETL----------------

CqRCC1_23 IYTYGWSKYGQLGHGDFE-D---------H--LTPYKLEA---LS-----

CqRCC1_24 VFIFGERRINKLNFGNSD-N---------V--TGPSMMSE---LP-----

CqRCC1_25 LFTWGDGDKGRLGHGDKE-A---------K--LVPTCVAD---LV-----

CqRCC1_26 VYTWGKGYCGALGHGDEI-E-----------KTTPGIVSS---LK-----

CqRCC1_27 VYGWGRGEHGRLGFGDDKSS-----------KMLPQKVQL---LA-----

CqRCC1_28 VYTWGYGGYGRLGHREQK-D---------E--WSPRRLDV---FT-----

CqRCC1_29 LFTWGEGDKGQLGHGDKE-A---------R--LIPECVEV---LM-----

CqRCC1_3 VYSWGWNGSS--

CqRCC1_30 LFTWGDGDKNRLGQGDKE-P---------R--LKPTCVPS---LI-----

CqRCC1_31 VYGFGSGKRGQLGVSEDK-I---------KSVSFPKTCSG---LE-----

CqRCC1_32 LFTWGDGDKGRLGHGDKE-A---------K--LVPTCVAA---LV-----

CqRCC1_33 LFTWGDGDKGRLGHGDKE-A---------K--LVPTCVAA---LV-----

CqRCC1_34 LFTWGDGDKFRLGQGNKD-A---------Y--LLPTCVSA---LI-----

CqRCC1_35 VYGWGRGEHGRLGFGDDKSS-----------KMLPQKVQL---LA-----

CqRCC1_36 --

CqRCC1_37 LFTWGEGDKGQLGHDDKE-A---------R--LIPECVEV---LM-----

CqRCC1_38 VCTWGWGRYGCLGHGNED-C-----------ESAPKVVEG---LT-----

CqRCC1_39 VLSWGHGGHG--

CqRCC1_4 VYTWGYGGYGRLGHREQK-D---------E--WSPRRLDV---FT-----

CqRCC1_40 VFTWGHRLVT------------------------PRRVIIARNLKKNGNA

CqRCC1_5 ---------------------------------------G---LE-----

CqRCC1_6 --

CqRCC1_7 VYTWGKGYCGALGHGDEI-E-----------KTTPGIVSS---LK-----

CqRCC1_8 LFTWGDGDKFRLGQGNKD-A---------Y--LLPTCVSA---LI-----

CqRCC1_9 VYSWGWNGSS--

CqRCC1_1 --

CqRCC1_10 --------EY-NF----HK-------------------------------

CqRCC1_11 --

CqRCC1_12 --------ENAYF----VS-------------------------------

CqRCC1_13 LMKFHRMERL-HV----VS-------------------------------

CqRCC1_14 --------VR-ES----CQLCGVYPGSEALCSGDTIICRSCVIELPFPLN

CqRCC1_15 --

CqRCC1_16 --------ENAYF----VS-------------------------------

CqRCC1_17 --------IPSGV----QL-------------------------------

CqRCC1_18 --------IPSGV----QL-------------------------------

CqRCC1_19 -----------IS----EE-------------------------------

CqRCC1_2 --------EV-KA----IH-------------------------------

CqRCC1_20 --------GQ-II----SQ-------------------------------

CqRCC1_21 --------EP-NF----CQ-------------------------------

CqRCC1_22 --

CqRCC1_23 --------GQ-MI----SQ-------------------------------

CqRCC1_24 -----------IS----EE-------------------------------

CqRCC1_25 --------EP-NF----CQ-------------------------------

CqRCC1_26 --------NH-LA----VQ-------------------------------

CqRCC1_27 --

CqRCC1_28 --------RH-NVLPSDAI-------------------------------

CqRCC1_29 --------DN-SF----CK-------------------------------

CqRCC1_3 --

CqRCC1_30 --------EY-NF----HK-------------------------------

CqRCC1_31 --------HA-EV----VA-------------------------------

CqRCC1_32 --------DP-NF----CQ-------------------------------

CqRCC1_33 --------DP-NF----CQ-------------------------------

CqRCC1_34 --------DY-SF----NQ-------------------------------

CqRCC1_35 --

CqRCC1_36 --

CqRCC1_37 --------DN-SF----CK-------------------------------

CqRCC1_38 --------DV-KA----IH-------------------------------

CqRCC1_39 --

CqRCC1_4 --------RH-NVLPSDAI-------------------------------

CqRCC1_40 LMKFHRMERL-HV----VS-------------------------------

CqRCC1_5 --------HA-EV----VA-------------------------------

CqRCC1_6 -----------Q--------------------------------------

CqRCC1_7 --------NH-LA----VQ-------------------------------

CqRCC1_8 --------DY-SF----NQ-------------------------------

CqRCC1_9 --

CqRCC1_1 --

CqRCC1_10 -----------------VACGHSLTVGLT----------------TMGHV

CqRCC1_11 --

CqRCC1_12 -----------------VDCGLLHTSVV----------------SSAGEV

CqRCC1_13 -----------------IATGMVHSMALT----------------DDG--

CqRCC1_14 SRINVEVGNVSQPVSSPVAISPSYSV------------------------

CqRCC1_15 --

CqRCC1_16 -----------------VDCGLLHTSVV----------------SSAGEV

CqRCC1_17 -----------------VACGHSHTLIS----------------TSDGRI

CqRCC1_18 -----------------VACGHSHTLIS----------------TSDGRI

CqRCC1_19 -----------------VACGGYHTCVVS----------------RHGEL

CqRCC1_2 -----------------VATGDYTTFVVS----------------DNGDV

CqRCC1_20 -----------------ISGGWRHTMAVT----------------SEGKL

CqRCC1_21 -----------------VTCGHSITVALT----------------TSGHV

CqRCC1_22 --

CqRCC1_23 -----------------ISGGWRHTMAVT----------------SEGKL

CqRCC1_24 -----------------VACGGYHTCVVS----------------RHGEL

CqRCC1_25 -----------------VTCGYSITVALT----------------TSGHV

CqRCC1_26 -----------------VCASKRKTYVLV----------------DTGSV

CqRCC1_27 --

CqRCC1_28 -----------------VSAGGVSSACTAGMALIYPSAELITDFRAGGQL

CqRCC1_29 -----------------VACGRDFTVALA----------------TSGRV

CqRCC1_3 --

CqRCC1_30 -----------------VACGHSLTVGLT----------------TMGHV

CqRCC1_31 -----------------ILANGDQSAALS----------------GSGRL

CqRCC1_32 -----------------VACGHSLTVALT----------------TTGQV

CqRCC1_33 -----------------VACGHSLTVALT----------------TTGQV

CqRCC1_34 -----------------IACGHNFTVALT----------------TSGHV

CqRCC1_35 --

CqRCC1_36 --

CqRCC1_37 -----------------VACGRDFTVALA----------------TSGRV

CqRCC1_38 -----------------VATGDYTTFVVS----------------DSGDV

CqRCC1_39 --

CqRCC1_4 -----------------VSAGGVSSACT----------------AAGGQL

CqRCC1_40 -----------------IATGMVHSMALT----------------DDG--

CqRCC1_5 -----------------ILANGDQSAALS----------------ASGQL

CqRCC1_6 --

CqRCC1_7 -----------------VCASKRKTYVLV----------------DTGSV

CqRCC1_8 -----------------IACGHNFTVALT----------------TSGHV

CqRCC1_9 --

CqRCC1_1 --------------------------------------PRLLDAP-SIES

CqRCC1_10 FTMGSTV-------YGQLGNP-F---------SDG-KLPCLVED--RLGG

CqRCC1_11 ----------------QLGHA-S---------IQNQKVPTMIE---ALAD

CqRCC1_12 WSWGMEKGLGLCPDVSFSGVD-H---------GDA-ILPLQFSCNGSNF-

CqRCC1_13 ----------------ALFYWIS---------SDPDLKCQQLC---YLNG

CqRCC1_14 --

CqRCC1_15 --

CqRCC1_16 WSWGMEKGLGLCPDVSFSGVD-H---------GDA-ILPLQFSCNGSNF-

CqRCC1_17 H---

CqRCC1_18 H---

CqRCC1_19 YAWGSNE-------NGCLGIG-C---------TDVIHSPERVEG--PFLR

CqRCC1_2 YSFGCGES-------SSLGHY-DGTDGEGNRHAN-VLKPELVT---SLKQ

CqRCC1_20 YGWGWNKF-------GQVGVGDN---------ADC-CSPVQ---------

CqRCC1_21 YTMGSPV-------YGQLGNP-H---------SDG-KLPTRVEG--KLLK

CqRCC1_22 --------------------------------------PRLLDAP-SIES

CqRCC1_23 YGWGWNK---

CqRCC1_24 YTWGSNE-------NGCLGIG-C---------TDVIHSPERVEG--PFLR

CqRCC1_25 YTMGSPV-------YGQLGNP-H---------SDG-KLPTRVEG--KLSK

CqRCC1_26 YAFGWMAF-------GSLGFT-DR------GASDKVTRPRILD---SLRG

CqRCC1_27 --

CqRCC1_28 YMWG------------KMKNT-G---------DDW-MYPKPVMDLS--G-

CqRCC1_29 YTMGSTA-------YGQLGCP-A---------ANE-RTPFCVEG--KIAD

CqRCC1_3 ----------------QLGRP-G---------HES--KPLAVE---SLAE

CqRCC1_30 FTMGSTV-------YGQLGNP-F---------SDG-KLPCLVED--RLGG

CqRCC1_31 YTWGRSF-------SSSS---------------NV-CIPQSVC-----SS

CqRCC1_32 YTMGSPV-------YGQLGNP-Q---------ADG-KLPVRVDG--KLVK

CqRCC1_33 YTMGSPV-------YGQLGNP-Q---------ADG-KLPVRVDG--KLVK

CqRCC1_34 FTMGSPS-------HGQLGNP-Q---------ADG-KSPSLVQD--KLIG

CqRCC1_35 --

CqRCC1_36 --

CqRCC1_37 YTMGSTA-------YGQLGCP-A---------ANG-RIPFCVEG--KIAD

CqRCC1_38 YSFGCGES-------SSLGHY-NGTDGEGNRHAN-VLKPELVT---SLKQ

CqRCC1_39 ----------------QLGHT-S---------IQNQKVPTMIE---ALAD

CqRCC1_4 YMWG------------KMKNT-G---------DDW-MYPKPVMDLS--G-

CqRCC1_40 ----------------ALFYWIS---------SDPDLKCQQLC---YLNG

CqRCC1_5 YTWGKSF-------SSSS---------------NV-CIPQLLR-----VS

CqRCC1_6 --

CqRCC1_7 YAFGWMAF-------GSLGFT-DR------GASDKVTRPRILD---SLRG

CqRCC1_8 FTMGSPS-------HGQLGNP-Q---------ADG-KSPSLVQD--KLIG

CqRCC1_9 ----------------QLGRP-G---------HES--KPLAVE---SLAE

```
CqRCC1_1     --VNAKIV-----SCGA-RHSAVVTEDG-KV--FCWGW------------
CqRCC1_10    --EMVEEI-----ACGA-YHVAVLTSRN-EV--YTWGK------------
CqRCC1_11    --ECVISI-----ACGG-SASAAVTATG-KL--FMWGN------------
CqRCC1_12    --PDPVEV-----ACG-AAHTVLVADDGYKL--WSWGR------------
CqRCC1_13    --KKIVSI-----SAGK-YWHGAVTSTG-DI--FMWDGK-----------
CqRCC1_14    ---------------------LSSPSNK-EKHRPLWAY------------
CqRCC1_15    --------------------------NY-TV--S----------------
CqRCC1_16    --PDPVEV-----ACG-AAHTVLVADDGYKL--WSWGR------------
CqRCC1_17    ----------------------------------GWGY------------
CqRCC1_18    ----------------------------------GWGY------------
CqRCC1_19    --QSVSKV-----SCGW-KHTAAIS-DG-YV--FTWGWGGSHGTFSVDGH
CqRCC1_2     VKERVVQISLTNSIFWN-AHTFALTESG-KL--YAFGA------------
CqRCC1_20    ---KVMQI-----CCGW-RHTLAVTERN-NV--FSWGR------------
CqRCC1_21    --CFVEEI-----ACGA-YHVAALTSRT-EV--YTWGK------------
CqRCC1_22    --VNAKIV-----SCGA-RHSAIVTEDG-KV--FCWGW------------
CqRCC1_23    ---KVMQI-----CCGW-RHTLAVTERN-NV--FSWGR------------
CqRCC1_24    --QSVSKV-----SCGW-KHTAAIS-DG-YV--FTWGWGGSHGTFSVDGH
CqRCC1_25    --CFVEEI-----ACGA-YHVAALTSRT-EV--YTWGK------------
CqRCC1_26    --HYISQI-----CTGL-YHTVVITNRG-QI--FGFGD------------
CqRCC1_27    --------------------------NE-DI--VQFGR------------
CqRCC1_28    --WNMRCM-----NSGQMFHY--VGADS-SC--ISWGH------------
CqRCC1_29    --NFVEDI-----ACGS-HHVAVLTSRA-EV--FTWGR------------
CqRCC1_3     --EIPTAV-----SCGR-VHSLAVTGNG-QL--WVWGC------------
CqRCC1_30    --ETVEEI-----ACGA-YHVAVLTSRN-EV--YTWGK------------
CqRCC1_31    --LSFEQA-----ALGW-NHALVLTSEG-EV--FMLGG------------
CqRCC1_32    --SFVEEI-----SCGA-YHVAALTSRT-EV--YTWGK------------
CqRCC1_33    --SFVEEI-----SCGA-YHVAALTSRT-EV--YTWGK------------
CqRCC1_34    --EYVEEI-----SCGA-HHVAVLTSRS-EV--YTWGK------------
CqRCC1_35    --------------------------NE-DI--VQFGR------------
CqRCC1_36    --------------------SAAVTEPG-EV--YGWGW------------
CqRCC1_37    --SFVEDI-----ACGS-HHVAVLTSKA-EV--FTWGR------------
```

CqRCC1_38 VKERIVQMSLTNSIFWN-AHTFALTESG-KL--YSFGA------------

CqRCC1_39 --ECVTSI-----ACGG-SASAAVTATG-KL--FMWGN------------

CqRCC1_4 --WNMRCM-----NSGQMFHY--VGADS-SC--ISWGH------------

CqRCC1_40 --KKIVSI-----SAGK-YWHGAVTSTG-DI--YMWDGK-----------

CqRCC1_5 --LSFEQA-----ALGW-NHALVLTSEG-EV--FMLGG------------

CqRCC1_6 ----ISLL-----SYGG-AHVIALTSGG-KV--LSWGR------------

CqRCC1_7 --HYISQI-----CTGL-YHTVVITNRG-QI--FGFGD------------

CqRCC1_8 --EYVEEI-----SCGA-HHVAVLTSRS-EV--YTWGK------------

CqRCC1_9 --EIPAAV-----SCGR-VHSLAVTGNG-QL--WVWGC------------

CqRCC1_1 NKYGQLG-------------------LG---DAID-----R-----NIPS

CqRCC1_10 GANGRLG-------------------HG--D-IED-----R-----KTPT

CqRCC1_11 AKDSQLG-------------------VP--G-LP------E-----INPS

CqRCC1_12 GRSGVLG-------------------DG--K-VSD-----S-----YSPT

CqRCC1_13 KAKDK---

CqRCC1_14 EIFGFVGIISGIFAFAYFIYLKRLGCSK--D-RKD-----R-----D---

CqRCC1_15 --

CqRCC1_16 GRSGVLG-------------------DG--K-VSD-----S-----YSPT

CqRCC1_17 NSYGQAS-------------------NE--K-STY-----A-----WYPS

CqRCC1_18 NSYGQAS-------------------NE--K-STY-----A-----WYPS

CqRCC1_19 SSGGQLG-------------------HG--T-DVDY----------IQPT

CqRCC1_2 GDKGQLG-------------------AKLGENQIE-----R-----GSPE

CqRCC1_20 GTNGQLG-------------------HG--D-SAD-----R-----NSPT

CqRCC1_21 GANGRLG-------------------HG--D-TED-----R-----NGPT

CqRCC1_22 NKYGQLG-------------------LG---DAID-----R-----NIPS

CqRCC1_23 GTNGQLG-------------------HG--D-SAD-----R-----NSPT

CqRCC1_24 SSGGQLG-------------------HG--T-DVDY----------IKPT

CqRCC1_25 GANGRLG-------------------HG--D-TED-----R-----NGPT

CqRCC1_26 NERAQLG-------------------LD--S-LRG-----C-----LEPT

CqRCC1_27 GDHGRLG-------------------YG--R-KVT-----T-----GHPM

CqRCC1_28 AQNGELG-------------------YG--P-NGQ-----KYTSSSANPK

CqRCC1_29 GTNGQLG-------------------HG--D-NDH-----R-----NEPT

CqRCC1_3 GKNGRLG-------------------LG--S-SFD-----E-----SEPA

CqRCC1_30 GANGRLG-------------------HG--D-VED-----R-----KTPT

CqRCC1_31 SHHGVLS-------------------EP--Q-KMTHLHNSS-----GEPT

CqRCC1_32 GANGRLG-------------------HG--D-TDD-----K-----NSPT

CqRCC1_33 GANGRLG-------------------HG--D-TDD-----K-----NSPT

CqRCC1_34 GANGRLG-------------------HG--D-VED-----R-----KSPT

CqRCC1_35 GDHGRLG-------------------YG--R-KVT-----T-----GHPM

CqRCC1_36 GRYGNLG-------------------LG--D-RKD-----R-----LVPE

CqRCC1_37 GTNGQLG-------------------HG--D-NDH-----R-----DEPT

CqRCC1_38 GDKGQLG-------------------AKLGENQIE-----R-----GSPE

CqRCC1_39 AKDSQLG-------------------VP--G-LP------E-----INPS

CqRCC1_4 AQNGELG-------------------YG--P-NGQ-----KYTSSSANPK

CqRCC1_40 KAKDK--

CqRCC1_5 SHHGVLS-------------------EP--Q-KMTHLHNTS-----GEPN

CqRCC1_6 GASGQLG-------------------LG--Q-IMPN----C-----LYPK

CqRCC1_7 NERAQLG-------------------LD--S-LRG-----C-----LEPT

CqRCC1_8 GANGRLG-------------------HG--D-VED-----R-----KFPT

CqRCC1_9 GKNGRLG-------------------LG--S-SFD-----E-----SEPA

CqRCC1_1 --QVKI------------DG--------------CVAK-KRGLWL-VAYT

CqRCC1_10 --LIEA---------L--KD-------------RHVKYITCGST-YTAA

CqRCC1_11 P--VEVNFLMEDDGLG--PH---------------NVLSVAVGAS-HALC

CqRCC1_12 --MVLWPP-------L--S------------------EDFSDGLN-I---

CqRCC1_13 P--PEVTRL-----HG--VK---------------RATKVSVGET-HLLI

CqRCC1_14 ----V----------V--KD--------------KELNSIGSSN------

CqRCC1_15 --------------------------------------SVVAGSR-NSLA

CqRCC1_16 --MVLWPP-------L--S------------------EDFSDGLN-I---

CqRCC1_17 --PVDW---------C--VG---------------EVRKLAAGGG-HSAV

CqRCC1_18 --PVDW---------C--VG---------------EVKKLAAGGG-HSAV

CqRCC1_19 --VIDV---------G--SN--------------VKAVE-----------

CqRCC1_2 --LVEI---------DL---------------------------------

CqRCC1_20 --MIEA---------L--SM--------------EG--------------

CqRCC1_21 --LVDA---------L--KD--------------KQVRSIACGTN-FTAA

CqRCC1_22 --QVKI------------DG--------------CVAKNVACGWW-HTLL

CqRCC1_23 --MIEA---------L--SM--------------EG--------------

CqRCC1_24 --VIDV---------G--SN--------------VKAVEVSCGFN-HTGA

CqRCC1_25 --LVEA---------L--KD--------------KQVRSIACGTN-FTAA

CqRCC1_26 --EIFV---------DEMMD--------------NAV-------------

CqRCC1_27 --EVPIN-----------LPPPRNDGEEEKAEARWRANLIACGGR-HTLA

CqRCC1_28 --KVEI---------L--EG--------------MHVISVACGFA-HAMV

CqRCC1_29 --LVEF---------M--KD--------------NQVKRVACGSN-FTAA

CqRCC1_3 L--VDI---------E--QY---------------KVVQAVAGTIQRLES

CqRCC1_30 --LIEA---------L--KD--------------RHVKYITCGST-YTAA

CqRCC1_31 LSKVTG---------L--DG--------------KKVAQIAAGAE-HSAI

CqRCC1_32 --LVEA---------L--KD--------------KQVKSIACGTN-FTAA

CqRCC1_33 --LVEA---------L--KD--------------KQVKSIACGTN-FTAA

CqRCC1_34 --MVEA---------L--RE--------------RLVKSVACGSN-LTAV

CqRCC1_35 --EVPIN-----------LPPPKSVGEEEKAEARWRANLIACGGR-HTLA

CqRCC1_36 --ISSS---------I--DV--------------C---------------

CqRCC1_37 --LVEF---------M--KD--------------NQVKRVACGSN-FTAA

CqRCC1_38 --LVEI---------DL---------------------------------

CqRCC1_39 P--VEVNFLMEDDGLG--PH---------------NVLSVAVGAS-HALC

CqRCC1_4 --KVEI---------L--EG--------------MHVISVACGFA-HAMV

CqRCC1_40 P--PEVTRL-----HG--VK---------------RATKVSVGET-HLLI

CqRCC1_5 LSKVTG---------L--DG--------------KKVVQIAAGAE-HSAI

CqRCC1_6 --LVDS---------L--TN--------------FNISHVSAGWN-HSGF

CqRCC1_7 --EIIV---------DEMMD--------------NAV-------------

CqRCC1_8 --MVEA---------L--RE--------------RLVKSVACGSN-LTAV

CqRCC1_9 L--VDI---------E--QY---------------KVVQAVAGFD-HTLL

CqRCC1_1 LTS---

CqRCC1_10 ICLHKWVSGAEQSQCSSCRQAFGFTRKRHNCYNCGLVHCHSCSSKKAFRA

CqRCC1_11 L---

CqRCC1_12 --

CqRCC1_13 V---------------GS--------TYHPA-------------------

CqRCC1_14 ---AAD----

CqRCC1_15 LCED--

CqRCC1_16 --

CqRCC1_17 LTN-------------------------------------ACSLKD----

CqRCC1_18 LTD-------------------------------------ACSLKD----

CqRCC1_19 --

CqRCC1_2 --

CqRCC1_20 --

CqRCC1_21 VCLHKWVSGVDQSMCSGCRLPFNFKRKRHNCYNCGLLFCHSCSSKKSFKA

CqRCC1_22 LAE---

CqRCC1_23 --

CqRCC1_24 IY------------------------------ECI---------------

CqRCC1_25 VCLHKWVSGVDQSMCSGCRLPFNFKRKRHNCYNCGLLFCHSCSSKKSFKA

CqRCC1_26 --

CqRCC1_27 IV--

CqRCC1_28 VVD-------------------------------------RTDVLD----

CqRCC1_29 ICLHKWASGTDHSTCTGCHNPFGFRRKRHNCYNCGQVFCKACSSRRSLKA

CqRCC1_3 I---

CqRCC1_30 ICLHKWVSGAEQSQCSSCRQAFGFTRKRHNCYNCGLVHCHSCSSKKAFRA

CqRCC1_31 LTGR--

CqRCC1_32 VCLHKWVSGVDQSLCSGCRVAFNFKKKRHNCYNCGLVFCHSCSSRKSLKA

CqRCC1_33 VCLHKWVSGVDQSLCSGCRVAFNFKKKRHNCYNCGLVFCHSCSSRKSLKA

CqRCC1_34 ICIHKWVSGADQSVCSGCRQAFGFTRKRHNCYNCGLVHCHACSSKKAVRA

CqRCC1_35 IVKW--

```
CqRCC1_36     --------------------------------------------------
CqRCC1_37     ICLHKWASGTDHSTCTGCHNPFGFRRKRHNCYNCGQVFCKACSSRRSLKA
CqRCC1_38     --------------------------------------------------
CqRCC1_39     L-------------------------------------------------
CqRCC1_4      VVD-------------------------------------RTDVGD----
CqRCC1_40     V---------------GS--------TYHPA-------------------
CqRCC1_5      LTGR------------------------------S-------NQAK----
CqRCC1_6      VSVV------------------------------G-------AR------
CqRCC1_7      --------------------------------------------------
CqRCC1_8      ICIHKWVSGADQSVCSGCRQAFGFTRKRHNCYNCGLVHCHACSSKKAVRA
CqRCC1_9      L-------------------------------------------------


CqRCC1_1      --------------------------------------------------
CqRCC1_10     ALAPNPSKPYRVCDTC---FAKLSKVLEGGSNR---GKSSG----PRLSG
CqRCC1_11     --------------------------------------------------
CqRCC1_12     --------------------------------------------------
CqRCC1_13     -------YPVNAVKDCENLSSAVSDV------------------------
CqRCC1_14     --------------SAFVASNASSAPQSRSSSITQLSS-MALGRTRS--W
CqRCC1_15     --------------------------------------------------
CqRCC1_16     --------------------------------------------------
CqRCC1_17     --------------L-----------------------------------
CqRCC1_18     --------------L-----------------------------------
CqRCC1_19     --------------------------------------------------
CqRCC1_2      --------------------------------------------------
CqRCC1_20     -------------------------------------S-GG----QRIEA
CqRCC1_21     SMAPNPNKPYRVCDNC---LTKLRKAVETGAST---RSSAS----RRGIV
CqRCC1_22     --------------------------------------------------
CqRCC1_23     -------------------------------------S-GG----QRIEA
CqRCC1_24     --------------------------------------------------
CqRCC1_25     SMAPNPNKPYRVCDNC---LTKLRKAVETGAST---RSSAS----RRGIV
```

CqRCC1_26 --

CqRCC1_27 --

CqRCC1_28 -------------R------------------------------------

CqRCC1_29 SLAPNMNKPYRVCDDC---YVKLKKATEYGNMR---IPRPV----SKISL

CqRCC1_3 --

CqRCC1_30 ALAPNPSKPYRVCDTC---FAKLSKVLEGGSNR---GKSSG----PRLSG

CqRCC1_31 --

CqRCC1_32 SMAPNPNKPYRVCDNC---FGKLRKSIENDSSS---ISSLS----RRGSM

CqRCC1_33 SMAPNPNKPYRVCDNC---FGKLRKSIENDSSS---ISSLS----RRGSM

CqRCC1_34 ALAPTPSKPHRVCDAC---YSKLRATASGNASVFNKRPTSA----PRRSM

CqRCC1_35 --

CqRCC1_36 --

CqRCC1_37 SLAPNMNKPYRVCDDC---YVKLKKATEYGNMR---IPRPV----SKISL

CqRCC1_38 --

CqRCC1_39 --

CqRCC1_4 -------------R------------------------------------

CqRCC1_40 -------YPVNAVQDCENLKPAVSDV------------------------

CqRCC1_5 --

CqRCC1_6 --

CqRCC1_7 --

CqRCC1_8 ALAPTPSKPHRVCDAC---YSKLRATASGNASFFNKRPTSA----PRRSM

CqRCC1_9 --

CqRCC1_1 --

CqRCC1_10 EN-K-DKLDKAELRLS-----KSMLPPNLDL-------------------

CqRCC1_11 --

CqRCC1_12 --

CqRCC1_13 ---------------------LGELDEGFM--------------------

CqRCC1_14 D--------N-SSKHVE----KAEV-----FTLSELAAATKNFSLENKIG

CqRCC1_15 --

CqRCC1_16 --
CqRCC1_17 --
CqRCC1_18 --
CqRCC1_19 --
CqRCC1_2 --
CqRCC1_20 S--------N-ADPSAG----RILV-------------------------
CqRCC1_21 NQ-GTEPNER-EEKLDSGSRGQLARYS---S-------------------
CqRCC1_22 --
CqRCC1_23 S--------N-ADPSAG----RILV-------------------------
CqRCC1_24 --
CqRCC1_25 NQ-GTELNER-EEKLDSGSRGQLARYS---S-------------------
CqRCC1_26 --
CqRCC1_27 --
CqRCC1_28 --
CqRCC1_29 QK-SNEGAEK-ETLLP-----KFGGLAFVNS-------------------
CqRCC1_3 --
CqRCC1_30 EN-K-DKLDKAELRLS-----KSMLPPNLDL-------------------
CqRCC1_31 --
CqRCC1_32 NQRGNQNFDN-EDKNGPQALVPLGKYSPMDS-------------------
CqRCC1_33 NQRGNQNFDN-EDKNGPQALVPLGKYSPMDS-------------------
CqRCC1_34 DG-TKERSERPESRSS-----KLLLTPTTEP-------------------
CqRCC1_35 --
CqRCC1_36 --
CqRCC1_37 QK-SNEGAEK-ETLLP-----KFGGLSFVNS-------------------
CqRCC1_38 --
CqRCC1_39 --
CqRCC1_4 --
CqRCC1_40 ----------------------LGELDEGFM-------------------
CqRCC1_5 --
CqRCC1_6 --
CqRCC1_7 --

CqRCC1_8 DG-TKERTERPESRSS-----KLLLSPTTEP-------------------

CqRCC1_9 --

CqRCC1_1 --

CqRCC1_10 ------------------IKQLDTKAAKQGKKAETFSLIQTSQTP-----

CqRCC1_11 --

CqRCC1_12 ----------------------ESKKPKEG--------------------

CqRCC1_13 ------------------YNDMESDNAKSSLKMEN-VGDKVVPSLKGLCE

CqRCC1_14 RGSFGTVYKGKLSSAGCEVAIKREEICARS--------------------

CqRCC1_15 --

CqRCC1_16 ----------------------ESKKPKEG--------------------

CqRCC1_17 ------------------CEF-RLAESVTL--------------------

CqRCC1_18 ------------------CEF-RLAESVTL--------------------

CqRCC1_19 --

CqRCC1_2 --

CqRCC1_20 --

CqRCC1_21 ------------------MGSSE-GRSKKNKKYEY-DSSRVSPIP-----

CqRCC1_22 --

CqRCC1_23 --

CqRCC1_24 --

CqRCC1_25 ------------------MDSSE-GRSKKNKKFEY-DSSRVSPIP-----

CqRCC1_26 --

CqRCC1_27 --

CqRCC1_28 ------------------LEQLDVHDGKAS--------------------

CqRCC1_29 ------------------LKRIDSDRTKGGAKLGK-VENSIFPTF-----

CqRCC1_3 --

CqRCC1_30 ------------------IKQLDTKAAKQGKKAETFSLIQTSQTP-----

CqRCC1_31 --

CqRCC1_32 ------------------FKETESVSFKRNKKLEF-NSSRVSPLP-----

CqRCC1_33 ------------------FKETESVSYKRNKKLEF-NSSRVSPLP-----

CqRCC1_34 ------------------VKYLEVKSQRYGGKLDSFSMVRASQVP-----

CqRCC1_35 --

CqRCC1_36 --

CqRCC1_37 ------------------LKRIDSDRSKGGAKPGK-VESSIFPTF-----

CqRCC1_38 --

CqRCC1_39 --

CqRCC1_4 ------------------LDQLDVHDGKAS--------------------

CqRCC1_40 ------------------YNDMESDNAKSGVKMEN-VGDKVVPSLKGLCE

CqRCC1_5 -------------------------------YVKF---------------

CqRCC1_6 --

CqRCC1_7 --

CqRCC1_8 ------------------VKYLEVKSQRYGGKLDSFSMVRASQVP-----

CqRCC1_9 --

CqRCC1_1 --

CqRCC1_10 --------------------------------------MLQ-LKDVVLSG

CqRCC1_11 --

CqRCC1_12 --

CqRCC1_13 KVAAQCLVEPRNALQMLEIADTLEAGDLRKYCEEIVIRNFDYI---FTAS

CqRCC1_14 --

CqRCC1_15 --

CqRCC1_16 --

CqRCC1_17 --

CqRCC1_18 --

CqRCC1_19 --

CqRCC1_2 --

CqRCC1_20 --

CqRCC1_21 --NGG---SQWG

CqRCC1_22 --

CqRCC1_23 --

```
CqRCC1_24    --------------------------------------------------
CqRCC1_25    ----------------------------------------NGG---SQWG
CqRCC1_26    --------------------------------------------------
CqRCC1_27    --------------------------------------------------
CqRCC1_28    --------------------------------------------------
CqRCC1_29    ----------------------------------------NGI---FQPG
CqRCC1_3     ----CCLEK-----------------------------------------
CqRCC1_30    --------------------------------------LLQ-LKDVVLSG
CqRCC1_31    --------------------------------------------------
CqRCC1_32    ----------------------------------------NGF---THWG
CqRCC1_33    ----------------------------------------NGF---THWG
CqRCC1_34    --------------------------------------TFQQLKDVAFPS
CqRCC1_35    --------------------------------------------------
CqRCC1_36    --------------------------------------------------
CqRCC1_37    ----------------------------------------NGI---FQPG
CqRCC1_38    --------------------------------------------------
CqRCC1_39    --------------------------------------------------
CqRCC1_4     --------------------------------------------------
CqRCC1_40    KVAAQCLVEPRNALQMLEIADTLDAGDLRKYCEEIVIRNFDYI---FTAS
CqRCC1_5     --------------------------------------------------
CqRCC1_6     --------------------------------------------------
CqRCC1_7     --------------------------------------------------
CqRCC1_8     --------------------------------------TFQQLKDVAFPS
CqRCC1_9     ----VAE-------------------------------------------


CqRCC1_1     --------------------------------------------------
CqRCC1_10    SADVKQTIPR---------PVATTAAATSISRPVSPFSRKPSPPRSATPV
CqRCC1_11    --------------------------------------------------
CqRCC1_12    --------------------------------------------------
CqRCC1_13    -LNTVATASLEVLAHIEKV---------LDVRSSESWSHR----RLPTPT
```

CqRCC1_14 --

CqRCC1_15 --

CqRCC1_16 --

CqRCC1_17 --

CqRCC1_18 --

CqRCC1_19 --

CqRCC1_2 --

CqRCC1_20 --

CqRCC1_21 -A-NISKSFNPMFGSSKKFFSASVPGSRIVSRATSPISRRPSPPRSTTPT

CqRCC1_22 --

CqRCC1_23 --

CqRCC1_24 --

CqRCC1_25 -A-NISKSFNPMFGSSKKFFSASVPGSRIVSRATSPISRRPSPPRSTTPT

CqRCC1_26 --

CqRCC1_27 --

CqRCC1_28 --

CqRCC1_29 -SSHVSSSLYPMVGTSDKRVSASAPGSRMVSRAASPISQGCSPARSFTFN

CqRCC1_3 --

CqRCC1_30 SADVKQTIPR---------PVATTAAATSISRPVSPFSRKPSPPRSATPV

CqRCC1_31 --

CqRCC1_32 -ALSTSK-----SGSSKKFFSASLPGSRIVSRATSPTSRRSSPPRAATPS

CqRCC1_33 -ALSTSK-----SGSSKKFFSASLPGSRIVSRATSPTSRRSSPPRAATPS

CqRCC1_34 SLSALQSALRPSTPTP---PPAPPVPSSLPARPSSPYTRRPSPPRSGTPV

CqRCC1_35 --

CqRCC1_36 --

CqRCC1_37 -SSHVSSSLYPMLGTSDKRVSASAPGSRMVSRAASPISQGCSPVRSFTFN

CqRCC1_38 --

CqRCC1_39 --

CqRCC1_4 --

CqRCC1_40 -LNTVATASLEILAHVEKV---------LDVRSSESWSHR----RLPTPT

CqRCC1_5 ---GSR

CqRCC1_6 --

CqRCC1_7 --

CqRCC1_8 SLSALQSALRPSTPTP---PPAPPVPSSLPARPSSPYTRRPSPPRSGTPV

CqRCC1_9 --

CqRCC1_1 --

CqRCC1_10 PTTS-GLSFSKS---------------IS-----------DSLKKTNELL

CqRCC1_11 --

CqRCC1_12 --

CqRCC1_13 ATFPAVLNSEEENSDSESPRTRD-NLSVTDSAKKAGCFRLDNFLQPMNVT

CqRCC1_14 --

CqRCC1_15 --

CqRCC1_16 --

CqRCC1_17 --

CqRCC1_18 --

CqRCC1_19 --

CqRCC1_2 --

CqRCC1_20 --

CqRCC1_21 PTLS-GLMSPKV---------------TV-----------DDAKGTNDSL

CqRCC1_22 --

CqRCC1_23 --

CqRCC1_24 --

CqRCC1_25 PTLS-GLMSPKV---------------TV-----------DDAKGTNDSL

CqRCC1_26 --

CqRCC1_27 --

CqRCC1_28 --

CqRCC1_29 NSIS-LPTSPEV---------------MA-----------PNSKPTQDDL

CqRCC1_3 --

CqRCC1_30 PTTS-GLSFSKS---------------IS-----------DSLKKTNELL

CqRCC1_31 --

CqRCC1_32 PTMV-VLSSPNK---------------VL-----------DDAKRTNDHL

CqRCC1_33 PTMV-VLSSPNK---------------VL-----------DDAKRTNDHL

CqRCC1_34 I--------SRG---------------IL-----------DSLKKSNELL

CqRCC1_35 --

CqRCC1_36 --

CqRCC1_37 NSIS-LPTSPEL---------------MA-----------PNSKPTQDNL

CqRCC1_38 --

CqRCC1_39 --

CqRCC1_4 --

CqRCC1_40 ATFPAVLNSEVENSDSESPRTRDNNLTVTYSAKKAGCFRLDNFLQPMNVT

CqRCC1_5 TVL---

CqRCC1_6 --

CqRCC1_7 --

CqRCC1_8 I--------SRG---------------IL-----------DSLKKSNELL

CqRCC1_9 --

CqRCC1_1 --

CqRCC1_10 NQEVVKLRAQVESLQQRCEL-QELELQKSSKKTQEAMALAADETAKTKAA

CqRCC1_11 --

CqRCC1_12 --------------GEKTE-VD-E---------------------KLDSA

CqRCC1_13 NQEVSK---KIRVLRKKL---QQIEV--LEAKQSKGHILDDQQIAKLE-T

CqRCC1_14 ---------------KRGYAFE-SEL-----------------MLLTRVH

CqRCC1_15 --

CqRCC1_16 --------------GEKTE-VD-E---------------------KLSSA

CqRCC1_17 --------------ANAAH-IEDVAF---------------------RTS

CqRCC1_18 --------------ANAAH-IEDVAF---------------------RTS

CqRCC1_19 --

CqRCC1_2 --

CqRCC1_20 --

CqRCC1_21 SQEVIRLRAQVESLTRKAQV-QEVELERANKQLKEAISIAGEETAKCKAA

CqRCC1_22 --

CqRCC1_23 --

CqRCC1_24 --

CqRCC1_25 SQEVIRLRAQVESLTRKAQV-QEVELERANKQLKEAISIAGEETAKCKAA

CqRCC1_26 --

CqRCC1_27 --

CqRCC1_28 --------------GEVAE-VE-TPVTSAKKTTKK---SPADNSKKRKKS

CqRCC1_29 RQEVVQLRVEVEKLTGDNRL-LEAELEKTRKRLKEAKVVAVDEAEKSKAA

CqRCC1_3 --

CqRCC1_30 NQEVVKLRAQVESLQQRCEL-QELELQKSSKKTQEAVALAADETAKTKAA

CqRCC1_31 --

CqRCC1_32 SQEVLKLRAQVEELTRKTQL-QDVELEKTSEQLTEAIAIAGEETAKCKAA

CqRCC1_33 SQEVLKLRAQVEELTRKTQF-QDVELEKTSKQLKEAIAIAGEETAKCKAA

CqRCC1_34 SQEVSKFQNHIKTLKQKCDY-QDDEIQKLQNNAKEASLLAADQSSKCKSA

CqRCC1_35 --

CqRCC1_36 --

CqRCC1_37 RQEIVQLRVEVEKLTGDNKL-LEAELEKTRKRLKEAKVVAVDEAEKSKAA

CqRCC1_38 --

CqRCC1_39 --

CqRCC1_4 --------------GEVTE-VE-TPVTSAKKPTKK---SPADNSKKRKKS

CqRCC1_40 NQEVSK---QIRVLRKKL---QQIEV--LEAKQSKGHILDDQQIAKLE-T

CqRCC1_5 --

CqRCC1_6 --

CqRCC1_7 --

CqRCC1_8 SQEVSKLQNHIKTLKQKCDY-QDDEIQKLQNSAREASLLATDQSSKWKYA

CqRCC1_9 --

CqRCC1_1 ---------RISHL------------------------------------

CqRCC1_10 KDVIKSLTAQLKDMAERL-----------------PPGAYDLENIKM-P-

CqRCC1_11 --

CqRCC1_12 MEEMKLLQSKLSVMERYVSILHGSLFAKPFEESDIP--------------
CqRCC1_13 RLALERSLAELGVPI-----------------------------------
CqRCC1_14 HKHLVGLVGFCEEQNERL-----------------LV-------------
CqRCC1_15 --
CqRCC1_16 MEEMKLLQSKLSVMESYVSVLHGSLFGKPFEESDIP--------------
CqRCC1_17 SDALVRLCGRLR--------------------------------------
CqRCC1_18 SDALVRLCGRLRELQ-----------------------------------
CqRCC1_19 --
CqRCC1_2 --
CqRCC1_20 --
CqRCC1_21 KEVIKSLTAQLKEMAERL-----------------PVGATRNSKSPVFP-
CqRCC1_22 ---------SP---------------------------------------
CqRCC1_23 --
CqRCC1_24 --
CqRCC1_25 KEVIKSLTAQLKEMAERL-----------------PVGATRNSKSPVFP-
CqRCC1_26 --
CqRCC1_27 --
CqRCC1_28 KDLGRG--
CqRCC1_29 KEVIKSLTAQLKEMAEKL-----------------QGGKDASSKLDF-C-
CqRCC1_3 --
CqRCC1_30 KDVIKSLTAQLKDMAERL-----------------PPGAYDLENIKM-P-
CqRCC1_31 --
CqRCC1_32 KEVIKSLTAQLKEMAERL-----------------PLGAVRNSKASV-P-
CqRCC1_33 KEVIKSLTAQLKEMAERL-----------------PLGAVRNSKVSV-P-
CqRCC1_34 RKAVESLSVQLKDIVEKM-----------------PSGANQSVNLQA-MR
CqRCC1_35 --
CqRCC1_36 --
CqRCC1_37 KEVIKSLTAQLKEMAEKL-----------------QGGKDASSKLDF-C-
CqRCC1_38 --
CqRCC1_39 --
CqRCC1_4 KDLSESEEEE--

CqRCC1_40 RPALESSLAELGVPI-----------------------------------

CqRCC1_5 --

CqRCC1_6 --

CqRCC1_7 --

CqRCC1_8 RKAVESFSVQLKDIVEKM-----------------PSEASQSENLRA-MR

CqRCC1_9 --

CqRCC1_1 --

CqRCC1_10 ---QL-PNGL

CqRCC1_11 --

CqRCC1_12 --

CqRCC1_13 -----------------------ETTIAKSSVSVVSDGKVSKAGM-PKKQ

CqRCC1_14 --

CqRCC1_15 --

CqRCC1_16 --

CqRCC1_17 --

CqRCC1_18 --

CqRCC1_19 --

CqRCC1_2 --

CqRCC1_20 --

CqRCC1_21 ---SLSST--

CqRCC1_22 --

CqRCC1_23 --

CqRCC1_24 --

CqRCC1_25 ---SLSST--

CqRCC1_26 --

CqRCC1_27 --

CqRCC1_28 --

CqRCC1_29 ---GQ-SS--

CqRCC1_3 --

CqRCC1_30 ---QL-PNGL

CqRCC1_31 --

CqRCC1_32 ---GS-PG--

CqRCC1_33 ---GS-PG--

CqRCC1_34 TQILAFLFTNETEASNSQTTTPVDLQALQKNQQSENKESTPQTSL-SVDL

CqRCC1_35 --

CqRCC1_36 --

CqRCC1_37 ---TQ-SS--

CqRCC1_38 --

CqRCC1_39 --

CqRCC1_4 --

CqRCC1_40 -----------------------ETTIAKSSVSVVSDGKVSKAGK-PKKQ

CqRCC1_5 --

CqRCC1_6 --

CqRCC1_7 --

CqRCC1_8 TQILAFLQTNETEASSSQTTIPVELQLQQKSQQSENKAPTPQTSL-PVDL

CqRCC1_9 --

CqRCC1_1 --

CqRCC1_10 EPNG--

CqRCC1_11 --

CqRCC1_12 --

CqRCC1_13 KRKSRQKESQTE------------------------------AYSGTELE

CqRCC1_14 -------------------------------------YKYMTNGALHDHL

CqRCC1_15 --

CqRCC1_16 --

CqRCC1_17 --

CqRCC1_18 --

CqRCC1_19 --

CqRCC1_2 --

CqRCC1_20 --

CqRCC1_21 --

CqRCC1_22 --

CqRCC1_23 --

CqRCC1_24 --

CqRCC1_25 --

CqRCC1_26 --

CqRCC1_27 --

CqRCC1_28 --

CqRCC1_29 --

CqRCC1_3 --

CqRCC1_30 EPNG--

CqRCC1_31 --

CqRCC1_32 --

CqRCC1_33 --

CqRCC1_34 QTQQQNQQVQNEASTNLPAEAQREQQSQQSEGNQVDDKSASAIQNSDQMS

CqRCC1_35 --

CqRCC1_36 --

CqRCC1_37 --

CqRCC1_38 --

CqRCC1_39 --

CqRCC1_4 --

CqRCC1_40 RRKSRHEESQTE------------------------------AYSGTELE

CqRCC1_5 --

CqRCC1_6 --

CqRCC1_7 --

CqRCC1_8 QPHQQNQQVQNEASTTFPVEAQLEQQSQQSEGNQVDDKSASTVQDSDKIS

CqRCC1_9 --

CqRCC1_1 --

CqRCC1_10 -------IH------------------------------------S-ADA

CqRCC1_11 --

CqRCC1_12 --

CqRCC1_13 SNHVKAVADLNLSKLTEGKISAEPEDSFTKGSQNLSQEKDSPGVGLSMFL

CqRCC1_14 HSYSAQKSSSPLNSWRMRIKIALDAARGIE--------------------

CqRCC1_15 --

CqRCC1_16 --

CqRCC1_17 --

CqRCC1_18 --

CqRCC1_19 --

CqRCC1_2 --

CqRCC1_20 --

CqRCC1_21 ---PTF-ADI

CqRCC1_22 --

CqRCC1_23 --

CqRCC1_24 --

CqRCC1_25 ---PTF-ADI

CqRCC1_26 --

CqRCC1_27 --

CqRCC1_28 --

CqRCC1_29 ---GSF-RKV

CqRCC1_3 --

CqRCC1_30 -------IH------------------------------------S-ADA

CqRCC1_31 --

CqRCC1_32 ---PNLSNDV

CqRCC1_33 ---PNLSNDV

CqRCC1_34 SSLKNLSLN----------------------------------PKI-LDD

CqRCC1_35 --

CqRCC1_36 --

CqRCC1_37 ---GSF-RKV

CqRCC1_38 --

CqRCC1_39 --
CqRCC1_4 --
CqRCC1_40 SNHVKVVADLNLSKLTEGKVPAEPEDSFTKGSQNLSLEKDSPGV------
CqRCC1_5 --
CqRCC1_6 --
CqRCC1_7 --
CqRCC1_8 -PPKIMSLN------------------------------------D-LPA
CqRCC1_9 --

CqRCC1_1 --
CqRCC1_10 SGEHVLR-SNPMNGPISSL-------------------TGVDSSMTNGTQ
CqRCC1_11 --
CqRCC1_12 ----------------------------ASLIESDSFD------------
CqRCC1_13 SGA---
CqRCC1_14 --------------YLHNYAVPPIIHRDIKS-----SNIL----------
CqRCC1_15 --
CqRCC1_16 ----------------------------ASLIESDSFD------------
CqRCC1_17 --
CqRCC1_18 --
CqRCC1_19 --
CqRCC1_2 --
CqRCC1_20 --
CqRCC1_21 SNPS--
CqRCC1_22 --
CqRCC1_23 --
CqRCC1_24 --
CqRCC1_25 SNPS--
CqRCC1_26 --
CqRCC1_27 --
CqRCC1_28 --

CqRCC1_29 SDD---

CqRCC1_3 --

CqRCC1_30 SGEHVLR-SDPTNGSISSL-------------------TGVDSSTTNGTQ

CqRCC1_31 --

CqRCC1_32 SNV---

CqRCC1_33 SNV---

CqRCC1_34 QCESAIQDSDQTPSSLKNLSLNPTILGDLPTS---GENAMQDSNLTPLST

CqRCC1_35 --

CqRCC1_36 --

CqRCC1_37 SDD---

CqRCC1_38 --

CqRCC1_39 --

CqRCC1_4 --

CqRCC1_40 --

CqRCC1_5 --

CqRCC1_6 --

CqRCC1_7 --

CqRCC1_8 SGESAIEDSDQISSSLKNLSLNPTILGDLPGS---GESAMQDSNLTPLST

CqRCC1_9 --

CqRCC1_1 --

CqRCC1_10 GPLQSPR---

CqRCC1_11 --

CqRCC1_12 --

CqRCC1_13 ------------------LDEPSKVVSPPPPPSPKCEGPAWGGSKVSKMS

CqRCC1_14 --

CqRCC1_15 --

CqRCC1_16 --

CqRCC1_17 --

CqRCC1_18 --

CqRCC1_19 ---

CqRCC1_2 ---

CqRCC1_20 ---

CqRCC1_21 ------------------IDQ----------------------------

CqRCC1_22 ---

CqRCC1_23 ---

CqRCC1_24 ---

CqRCC1_25 ------------------INQ----------------------------

CqRCC1_26 ---

CqRCC1_27 ---

CqRCC1_28 ---

CqRCC1_29 ---

CqRCC1_3 ---

CqRCC1_30 GPLQSPR--

CqRCC1_31 ---

CqRCC1_32 ------------------MDR----------------------------

CqRCC1_33 ------------------MDR----------------------------

CqRCC1_34 DATETPRHTLEDRGQYSTIIQ----------------------------

CqRCC1_35 ---

CqRCC1_36 ---

CqRCC1_37 ---

CqRCC1_38 ---

CqRCC1_39 ---

CqRCC1_4 ---

CqRCC1_40 ------------------------------------GPAWGGSKVSKMS

CqRCC1_5 ---

CqRCC1_6 ---

CqRCC1_7 ---

CqRCC1_8 DTTETPRYNLEDGVQYSSVMQ----------------------------

CqRCC1_9 ---

CqRCC1_1 --

CqRCC1_10 --

CqRCC1_11 --

CqRCC1_12 --

CqRCC1_13 ASLREIQNEQSKLFNNLPTKSKEKPEELADRTSGNFLLSSLLASNPIPVV

CqRCC1_14 --

CqRCC1_15 --

CqRCC1_16 --

CqRCC1_17 --

CqRCC1_18 --

CqRCC1_19 --

CqRCC1_2 --

CqRCC1_20 --

CqRCC1_21 --

CqRCC1_22 --

CqRCC1_23 --

CqRCC1_24 --

CqRCC1_25 --

CqRCC1_26 --

CqRCC1_27 --

CqRCC1_28 --

CqRCC1_29 --

CqRCC1_3 --

CqRCC1_30 --

CqRCC1_31 --

CqRCC1_32 --

CqRCC1_33 --

CqRCC1_34 --

CqRCC1_35 --

CqRCC1_36 --

CqRCC1_37 --
CqRCC1_38 --
CqRCC1_39 --
CqRCC1_4 --
CqRCC1_40 SSLREIQNEQSKLFNNRPLKNKEKPEELPDRSSGKFLLSSLLASNPIPVA
CqRCC1_5 --
CqRCC1_6 --
CqRCC1_7 --
CqRCC1_8 --
CqRCC1_9 --

CqRCC1_1 --
CqRCC1_10 --------------------------------ESY------MLNENSYQL
CqRCC1_11 --
CqRCC1_12 ----------------------------------VGKA------------
CqRCC1_13 PARTTQLPDGDKSTPPWAASGTP-PHLSRPSLRDIQ------MQQGKNQS
CqRCC1_14 ----------------------------------LDAN------------
CqRCC1_15 --
CqRCC1_16 ----------------------------------VGKA------------
CqRCC1_17 --
CqRCC1_18 --
CqRCC1_19 --
CqRCC1_2 --
CqRCC1_20 --
CqRCC1_21 --------CNDQIPSLD----------------SYGFN------------
CqRCC1_22 --
CqRCC1_23 --
CqRCC1_24 --
CqRCC1_25 --------CNDQIPSLD----------------SYGFN------------
CqRCC1_26 --

CqRCC1_27 --

CqRCC1_28 --

CqRCC1_29 --

CqRCC1_3 --

CqRCC1_30 --------------------------------ESY------MLNENSYQL

CqRCC1_31 --

CqRCC1_32 --------LNSPV-AVH----------------EIGLN------------

CqRCC1_33 --------LNSPV-AVH----------------EIGLN------------

CqRCC1_34 --------DSDQAPTSLDTTETT-SHESENGVQDS------SVNQDSNQT

CqRCC1_35 --

CqRCC1_36 --

CqRCC1_37 --

CqRCC1_38 --

CqRCC1_39 --

CqRCC1_4 --

CqRCC1_40 PARTTQLPDGDKSTPPWAASGTP-PHLSRPSLRDIQ------MQQGKNQS

CqRCC1_5 --

CqRCC1_6 --

CqRCC1_7 --

CqRCC1_8 --------DSDQVPSSHDTTETTTSQDSENGVQDFSVNQDSSVNQDFNQT

CqRCC1_9 --

CqRCC1_1 --

CqRCC1_10 NESFAALKGREDQLHAKL------SAAAGGLQTCSSSVSDATDARFSASS

CqRCC1_11 --

CqRCC1_12 ---WQGMLEA

CqRCC1_13 ---------------------------------VAH--SPKTKTT-GFAV

CqRCC1_14 ---WVG-RVS

CqRCC1_15 --

CqRCC1_16 ---WQGMLEA

CqRCC1_17 --
CqRCC1_18 --
CqRCC1_19 --
CqRCC1_2 --
CqRCC1_20 --
CqRCC1_21 ---G---LMF
CqRCC1_22 --
CqRCC1_23 --
CqRCC1_24 --
CqRCC1_25 ---G---LVF
CqRCC1_26 --
CqRCC1_27 --
CqRCC1_28 --
CqRCC1_29 --
CqRCC1_3 --
CqRCC1_30 NESFAALKGREDQLHAKL------SAAAGGLQTCSSSVSDATDARFSASS
CqRCC1_31 --
CqRCC1_32 ---WSNRHDS
CqRCC1_33 ---WSNRHDS
CqRCC1_34 SPSSDTTEA--DTRHDSK------DEVRDPIQTPSS--SDTTDTTTKHDS
CqRCC1_35 --
CqRCC1_36 --
CqRCC1_37 --
CqRCC1_38 --
CqRCC1_39 --
CqRCC1_4 --EQDS
CqRCC1_40 ---------------------------------VAH--SPKTKTT-GFAV
CqRCC1_5 --
CqRCC1_6 --
CqRCC1_7 --
CqRCC1_8 SPSSDTTET--ATRHDSKDEVQDYSVKQDPIQTPSS--SDTTDTTTKHDS

CqRCC1_9 --

CqRCC1_1 --
CqRCC1_10 QDGFNSSRSKSPVP------------------------------------
CqRCC1_11 --
CqRCC1_12 AD--
CqRCC1_13 TTDQG---
CqRCC1_14 DF--
CqRCC1_15 --
CqRCC1_16 AD--
CqRCC1_17 --
CqRCC1_18 --
CqRCC1_19 --
CqRCC1_2 --
CqRCC1_20 --
CqRCC1_21 SNGSATPSS-----------------------------RSSVQSRVASTE
CqRCC1_22 --
CqRCC1_23 --
CqRCC1_24 --
CqRCC1_25 SNGSGTPSS-----------------------------RSSVQSRVASTE
CqRCC1_26 --
CqRCC1_27 --
CqRCC1_28 ----------------------------------RPSTKSPV--------
CqRCC1_29 --NFLSPRS-----------------------------EST---VTMDA-
CqRCC1_3 --
CqRCC1_30 QDGFNSCRSKSPVP------------------------------------
CqRCC1_31 --
CqRCC1_32 SNGSTTPSS-----------------------------RASNNLVTGQLE
CqRCC1_33 SNGSTTPSS-----------------------------RASNNLVTGQLE
CqRCC1_34 EDGTQEAS-EHPLSNEAESSANERSEENGGVQE---PNQSPS----PTTE

CqRCC1_35 --

CqRCC1_36 --

CqRCC1_37 --NFLSPRS----------------------------EST---VTMDA-

CqRCC1_38 --

CqRCC1_39 --

CqRCC1_4 DDGSDDQS-EEEMENG-HMDRRQRGGKAGGRGRGRPSTKSPG--------

CqRCC1_40 TTGQG---

CqRCC1_5 --

CqRCC1_6 --

CqRCC1_7 --

CqRCC1_8 EDGAQEAS-EHPLSNEAESSANERSEENGGVQE---PNQSLS----PTTE

CqRCC1_9 --

CqRCC1_1 --

CqRCC1_10 --

CqRCC1_11 --

CqRCC1_12 ----------------GKELRRLEMFYG---------------------

CqRCC1_13 --

CqRCC1_14 ----------------GLSLKGPENEEGSMSIKPVGTVGYIDPEYYVLKV

CqRCC1_15 --

CqRCC1_16 ----------------GKELRRLEMFYG---------------------

CqRCC1_17 --

CqRCC1_18 --

CqRCC1_19 --

CqRCC1_2 --

CqRCC1_20 --

CqRCC1_21 AT-RN-GNR---------------------------------------

CqRCC1_22 --

CqRCC1_23 --

CqRCC1_24 --

CqRCC1_25 AT-RN-GNR---

CqRCC1_26 --

CqRCC1_27 --

CqRCC1_28 ---------------ETKSIGRGR-GRP----------------------

CqRCC1_29 -LL---

CqRCC1_3 --

CqRCC1_30 --

CqRCC1_31 --

CqRCC1_32 ASCKN-KNR---

CqRCC1_33 ASCKN-KNR---

CqRCC1_34 FT-PNQGNKSAASKLS----------------------------------

CqRCC1_35 --

CqRCC1_36 --

CqRCC1_37 -LL---

CqRCC1_38 --

CqRCC1_39 --

CqRCC1_4 ---------------ETKSSGRGR-GRP----------------------

CqRCC1_40 --

CqRCC1_5 --

CqRCC1_6 --

CqRCC1_7 --

CqRCC1_8 LT-PNHGNNIAASKLS----------------------------------

CqRCC1_9 --

CqRCC1_1 --

CqRCC1_10 ------------------------SAANNNNQV-EAEWIEQY-EPG-----

CqRCC1_11 --

CqRCC1_12 ---------------NMLDGVK--------------------------D-

CqRCC1_13 -------------------------SPSESGA-QSRWFKPEVEST-----

CqRCC1_14 LTTKSDVYGFGVVLLELLTGKKAVFKEGEK-SGPTGVVEYA-GPFIVAGE

CqRCC1_15 --

CqRCC1_16 ---------------NMLDGVK--------------------------D-

CqRCC1_17 --

CqRCC1_18 ---------------------C--------------------------AG

CqRCC1_19 --

CqRCC1_2 --

CqRCC1_20 ----PPSGRYAVVPDEQASTAGSRDNGGDA--------------------

CqRCC1_21 ------------------------TREGEPRP-ENEWVEQD-EPG-----

CqRCC1_22 --

CqRCC1_23 ----PPSGRYAVVPDEQASTAGSRDNGGDA--------------------

CqRCC1_24 --

CqRCC1_25 ------------------------TREGEPRP-ENEWVEQD-EPG-----

CqRCC1_26 --

CqRCC1_27 --

CqRCC1_28 ------------------KG------------------------------

CqRCC1_29 ------------------------TNGEKAQPEKTEWVVQD-EPG-----

CqRCC1_3 --

CqRCC1_30 -----------------------SAANNNSQV-EAEWIEQY-EPG-----

CqRCC1_31 --

CqRCC1_32 ------------------------TSDSEANY-GSEWVEQD-EPG-----

CqRCC1_33 ------------------------TSDSEANY-GSEWVEQD-EPG-----

CqRCC1_34 -----------------------STSRNEGSG-SDERTEQI-ERG-----

CqRCC1_35 --

CqRCC1_36 --

CqRCC1_37 ------------------------TNGEKAQPEKTEWVVQD-EPG-----

CqRCC1_38 --

CqRCC1_39 --

CqRCC1_4 ------------------KG------------------------------

CqRCC1_40 -------------------------SPSESGS-QSRWFKPEVEST-----

CqRCC1_5 --

CqRCC1_6 --

CqRCC1_7 --

CqRCC1_8 -----------------------STSRN--EG-SDERTEQI-ERG-----

CqRCC1_9 --

CqRCC1_1 --

CqRCC1_10 ---------V--

CqRCC1_11 --

CqRCC1_12 KIMKRKIQEI--------VREH-----------LQSS-------------

CqRCC1_13 ---------S--

CqRCC1_14 VW-KVLDKRVEMPVMNESEAVELLAYTAMMCVNLEGKERPTMSDVVSNLE

CqRCC1_15 --

CqRCC1_16 KIMKRKIQEI--------VKEH-----------LQSS-------------

CqRCC1_17 --

CqRCC1_18 GWESEDDGSG--

CqRCC1_19 --

CqRCC1_2 --

CqRCC1_20 --

CqRCC1_21 ---------V--

CqRCC1_22 --

CqRCC1_23 --

CqRCC1_24 --

CqRCC1_25 ---------V--

CqRCC1_26 --

CqRCC1_27 --

CqRCC1_28 -------SRG--

CqRCC1_29 ---------V--

CqRCC1_3 --

CqRCC1_30 ---------V--

CqRCC1_31 --

CqRCC1_32 ---------V--

CqRCC1_33 ---------V--

CqRCC1_34 ---------V--

CqRCC1_35 --

CqRCC1_36 --

CqRCC1_37 ---------V--

CqRCC1_38 --

CqRCC1_39 --

CqRCC1_4 -------SRG--

CqRCC1_40 ---------S--

CqRCC1_5 ---------S--

CqRCC1_6 --

CqRCC1_7 --

CqRCC1_8 ---------V--

CqRCC1_9 --

CqRCC1_1 --------S-----------------------------WGT---------

CqRCC1_10 -YITLVALR-DGTRDLKRVRFSRRRFGEHQAE-----TWWSENREKVYER

CqRCC1_11 --------------------------VSRSSCTE----------------

CqRCC1_12 -----------------------------STT-----QY-----------

CqRCC1_13 -SIRSIQIEEKAIKDFRRF-YSSVRIVKNQS-------------------

CqRCC1_14 RALTLCKGS-SGTFSPNFYSFSEI-----IG-------------------

CqRCC1_15 ---------------------------------------GK---------

CqRCC1_16 -----------------------------STT-----QY-----------

CqRCC1_17 --

CqRCC1_18 -----------------------------LIR------P-----------

CqRCC1_19 --

CqRCC1_2 --

CqRCC1_20 ------SVP---ENDVKRIRVGPG-----PA-------------------

CqRCC1_21 -YITFISLP-GGIKDLKRVRFSRKRFSEKQAE-----QWWAANRERVYER

CqRCC1_22 --T---------

CqRCC1_23 ------SVP---ENDVKRIRVGPG-----PA-------------------

CqRCC1_24 --

CqRCC1_25 -YITFVSLP-GGIKDLKRVRFSRKRFSEKQAE-----QWWAANRERVYER

CqRCC1_26 --

CqRCC1_27 --

CqRCC1_28 -----------------------------KAR-----K------------

CqRCC1_29 -YITLCSLP-GGTNELKRLRFSNFVFSAANVSVKDKRKSGGLKTAAEYVN

CqRCC1_3 -----------SIG-YR---------------------------------

CqRCC1_30 -YITLVALR-DGTRDLKRVRFSRRRFGEHQAE-----TWWSENREKVYER

CqRCC1_31 --

CqRCC1_32 -YITLVSMP-DGIKDLKRVRFSRKRFSEKEAE-----QWWAANRARIYQQ

CqRCC1_33 -YITLVSMP-DGIKDLKRVRFSRKRFSEKEAE-----QWWAANRARIYQQ

CqRCC1_34 -YITYLELS-NGTKIFKRVRFSKKLFVATQAE-----DWWRENRERVYKE

CqRCC1_35 ---------------------------------------QADD-DEL---

CqRCC1_36 --

CqRCC1_37 -YITLCSLP-GGTNELKRLRFSRKRFSEGQAE-----KWWTENSSRVCER

CqRCC1_38 --

CqRCC1_39 --------------------------VSRSSCKE----------------

CqRCC1_4 -----------------------------KAR-----K------------

CqRCC1_40 -SIRSIQIEEKAIKDFRRF-YSSVRIVKNQS-------------------

CqRCC1_5 -YVVLISHD-HHLR-----------------------------A---KQT

CqRCC1_6 ----ALRVP-PTAK------------------------------------

CqRCC1_7 --

CqRCC1_8 -YITYLELP-NGTKIFKRVRFSKKLYVATQAE-----DWWRENREKVYRE

CqRCC1_9 ----------------S---------------------------------

CqRCC1_1 --

CqRCC1_10 YNVRGSDKSSVSGQTARRS---DGAMSPA--SPYN---------------

CqRCC1_11 --

CqRCC1_12 --

CqRCC1_13 --

CqRCC1_14 --

CqRCC1_15 --

CqRCC1_16 --

CqRCC1_17 --

CqRCC1_18 --

CqRCC1_19 --

CqRCC1_2 --------SS--

CqRCC1_20 --

CqRCC1_21 YNVRTSDKSSVGVES--------------DLGSFITDK------------

CqRCC1_22 --

CqRCC1_23 --

CqRCC1_24 --

CqRCC1_25 YNVRTSDKSSVGVES--------------DLGSFITDK------------

CqRCC1_26 -----ADKANV---------------------------------------

CqRCC1_27 --

CqRCC1_28 --

CqRCC1_29 GTTYRVNKCGLSVYY--------------HLGSKAGNNDSLIWSVLTWFL

CqRCC1_3 --

CqRCC1_30 YNVRGSDKSSVSGQTARRS---DGAMSPA--SSYN---------------

CqRCC1_31 --

CqRCC1_32 YDVPMVDKAGIGIGR--------------EGIAH----------------

CqRCC1_33 YDVPMVDKAGIGIGR--------------EGIAH----------------

CqRCC1_34 FIPAPPNSV-KPSRTSLPTAATSGLPPPNEEVCNA-----------NNAA

CqRCC1_35 --

CqRCC1_36 --

CqRCC1_37 HNIQSQ--

CqRCC1_38 --------SS--

CqRCC1_39 --

CqRCC1_4 --

CqRCC1_40 --

CqRCC1_5 Y--

CqRCC1_6 ---

CqRCC1_7 -----ADKANV------------------------------------

CqRCC1_8 FIPAPPNSVKPSSRTSLSKAATSSLPPPNEEDSNA-----------NNAA

CqRCC1_9 ---

CqRCC1_1 ----

CqRCC1_10 ----

CqRCC1_11 ----

CqRCC1_12 ----

CqRCC1_13 ----

CqRCC1_14 ----

CqRCC1_15 ----

CqRCC1_16 ----

CqRCC1_17 ----

CqRCC1_18 ----

CqRCC1_19 ----

CqRCC1_2 ----

CqRCC1_20 ----

CqRCC1_21 ----

CqRCC1_22 ----

CqRCC1_23 ----

CqRCC1_24 ----

CqRCC1_25 ----

CqRCC1_26 ----

CqRCC1_27 ----

CqRCC1_28 ----

CqRCC1_29 R---

CqRCC1_3 ----

CqRCC1_30 ----

CqRCC1_31	----
CqRCC1_32	----
CqRCC1_33	----
CqRCC1_34	P---
CqRCC1_35	----
CqRCC1_36	----
CqRCC1_37	----
CqRCC1_38	----
CqRCC1_39	----
CqRCC1_4	----
CqRCC1_40	----
CqRCC1_5	----
CqRCC1_6	----
CqRCC1_7	----
CqRCC1_8	SLET
CqRCC1_9	----
